# Optimal Bayesian design for model discrimination via classification

**DOI:** 10.1007/s11222-022-10078-2

**Published:** 2022-02-22

**Authors:** Markus Hainy, David J. Price, Olivier Restif, Christopher Drovandi

**Affiliations:** 1 Department of Applied Statistics, Johannes Kepler University, 4040 Linz, Austria; 2 School of Mathematical Sciences, Queensland University of Technology, Brisbane, QLD 4000 Australia; 3 Centre for Epidemiology and Biostatistics, Melbourne School of Population and Global Health, The University of Melbourne, Melbourne, VIC 3010 Australia; 4 The Department of Infectious Diseases at The Peter Doherty Institute for Infection and Immunity, The University of Melbourne and Royal Melbourne Hospital, Melbourne, VIC 3000 Australia; 5 Department of Veterinary Medicine, University of Cambridge, Cambridgeshire, CB3 0ES United Kingdom; 6 ARC Centre of Excellence for Mathematical & Statistical Frontiers, Melbourne, Australia; 7 QUT Centre for Data Science, Brisbane, Australia

**Keywords:** Approximate Bayesian computation, Bayesian model selection, Classification and regression tree, Continuous-time Markov process, Random forest, Simulation-based Bayesian experimental design

## Abstract

Performing optimal Bayesian design for discriminating between competing models is computationally intensive as it involves estimating posterior model probabilities for thousands of simulated data sets. This issue is compounded further when the likelihood functions for the rival models are computationally expensive. A new approach using supervised classification methods is developed to perform Bayesian optimal model discrimination design. This approach requires considerably fewer simulations from the candidate models than previous approaches using approximate Bayesian computation. Further, it is easy to assess the performance of the optimal design through the misclassification error rate. The approach is particularly useful in the presence of models with intractable likelihoods but can also provide computational advantages when the likelihoods are manageable.

**Supplementary Information:**

The online version contains supplementary material available at 10.1007/s11222-022-10078-2.

## Introduction

In many applications, finding the most appropriate model among a class of possible models is an important goal of statistical inference. In the classical literature, these decisions are commonly based on model selection criteria such as the Akaike information criterion or related criteria (Konishi and Kitagawa 2008). The Bayesian approach, where the model indicator is regarded as an additional unknown random variable, offers a coherent decision-theoretic framework for inference and model discrimination (Key et al. 1999). Common options to carry out model selection in a Bayesian context are Bayes factors (Kass and Raftery 1995), the deviance information criterion (Spiegelhalter et al. 2002), or the computation of the marginal likelihoods or evidence (Friel and Pettitt 2008). Given the prior model probabilities, the marginal likelihoods can be turned into posterior model probabilities. Classical model selection criteria only provide a ranking of the models, whereas posterior model probabilities contain useful information about the relative likeliness of the various models as well. In addition, the posterior model probabilities permit model-averaged predictions.

Prior to conducting an experiment, it is pertinent to determine the optimal combination of the controllable factors so as to maximise the (expected) information gain of the experiment with respect to the desired statistical objective (e.g. parameter inference, model discrimination, prediction). This is achieved by applying the principles and methods of optimal experimental design (see, e.g. Atkinson et al. 2007). In optimal experimental design, one seeks to find the optimal combination of the controllable factors in order to maximise the (expected) information gain of the experiment (Atkinson et al. 2007). If the main goal of statistical inference is to determine which statistical process is the most suitable representation of the phenomenon of interest, it is pertinent to employ design criteria specifically developed for the purpose of model discrimination. For example, in epidemiology it is paramount to understand the transmission dynamics of a disease in order to be able to implement effective countermeasures (see, e.g. Dehideniya et al. 2018a). The most commonly used classical design criterion is T-optimality (Atkinson and Federov 1975a, b; Dette and Titoff 2009), with extensions to Bayesian T-optimality (Ponce de Leon and Atkinson 1992) to incorporate prior information. Except for robust T-optimal designs (Vajjah and Duffull 2012), one model has to be selected as the assumed true model. For classical T-optimality, one seeks to maximise the $L_2$-norm of the difference between the assumed true model’s predictor and the other model’s predictor with respect to the design measure, where for each design the most unfavourable parameter setting with respect to the predictor difference is chosen for the second model. Therefore, T-optimal designs are generally computationally expensive. Harman and Müller (2020) propose a symmetric criterion based on the linearised distance between the mean value surfaces of the models, which can be computed quickly. Their designs depend on the set of parameters over which the criterion is optimised, so they suggest to consider different parameter set sizes and to choose the size of the set based on ones “confidence” about the true parameter value.

Fully Bayesian experimental design provides a consistent framework to handle parameter and model uncertainty when planning the experiment (Chaloner and Verdinelli 1995; Ryan et al. 2016). For model discrimination, the most popular design criterion is the mutual information between the model indicator and the data, which is measured by the Kullback–Leibler divergence between the joint and marginal distributions of those two random variables (see Box and Hill 1967). This criterion requires the computation of the evidence of each model for many potential observations, so its use has been confined to a limited set of applications such as simple models with conjugate priors (Ng and Chick 2004), cases where numerical quadrature is feasible (Cavagnaro et al. 2010), or sequential design settings (Drovandi et al. 2014). Overstall et al. (2018) employ normal-based approximations to find optimal designs for several criteria including mutual information and misclassification error. For the case of intractable likelihoods, Dehideniya et al. (2018b) use approximate Bayesian computation (ABC) to estimate these criteria. The ABC approach only requires the ability to simulate from all the candidate models. However, their approach is simulation- and memory-intensive and is thus limited to low-dimensional designs. Overstall and McGree (2019) propose an approach based on auxiliary models, whereas Dehideniya et al. (2018a) employ synthetic likelihoods. An extension of Overstall et al. (2018) for models with intractable likelihoods is developed by Dehideniya et al. (2019). Kleinegesse and Gutmann (2019) develop a design approach based on likelihood-free inference by ratio estimation (see Thomas et al. 2022), which is suitable for the commonly used mutual information-based design criteria, with an extension to sequential designs in Kleinegesse et al. (2020). Another approach for mutual information-based criteria, which can also be applied to intractable likelihood models, is presented by Foster et al. (2019), who use amortised variational inference to find an approximation to the posterior distribution that is part of the criterion.

Like Dehideniya et al. (2018b), we suggest a simulation-based approach. However, we use the outputs of standard supervised classification procedures from machine learning (see Hastie et al. 2009) to estimate the design criteria. In particular, we employ classification trees (Breiman et al. 1984) and random forests (Breiman 2001). We demonstrate that this approach considerably reduces the required number of simulations compared to ABC. In order to keep the computational burden manageable, Dehideniya et al. (2018b) pre-simulate a large sample from the prior predictive distribution at a grid of possible design points and reuse these simulations for all the designs they consider during the optimisation process, refining the grid over time. However, as we require fewer simulations for the classification approach, it is not necessary to pre-simulate the data. As a consequence, the classification approach is much more flexible and suitable for much higher-dimensional designs. Furthermore, the classification approach does not require direct approximations of posterior quantities such as the posterior model probabilities, which may only be reliably estimated with great computational effort, making it a viable alternative for many models with tractable likelihoods. Another advantage of the classification approach is that one can readily use the output from the classification procedures to assess the designs by estimating misclassification error rates or misclassification matrices. Our method represents a novel approach using supervised learning methods for optimal Bayesian design for model discrimination.

Section 2 reviews Bayesian experimental design and the associated expected utility and loss functions. Our classification approach is presented in Sect. 3 along with a discussion of classification and regression trees (CART) and random forests. In Sect. 4, we provide three examples to demonstrate the utility of the classification approach: discriminating between the epidemiological Markov process models of the same type as considered by Dehideniya et al. (2018b) (Sect. 4.1), a two-model variation of the previous example to be able to make comparisons with likelihood-based designs and apply our method to higher-dimensional settings (Sect. 4.2), and discriminating between three Markov process models describing the dynamics of bacteria within phagocytic cells (Sect. 4.3). The supplementary document (Online Resource 1) contains further details on CART and random forests, a description of the variant of the coordinate exchange algorithm that we employ for all our examples, a comparative investigation of the computational performances of the different methods for the three examples in Sect. 4, detailed model descriptions and further results for the three examples in Sect. 4, and two additional examples. The first additional example is a logistic regression example which has been considered for Bayesian experimental design before (e.g. Overstall et al. 2018); the second is about discriminating between three spatial extremes models for which Lee et al. (2018) perform ABC model discrimination for a given design.

## Optimal Bayesian design for model discrimination

We assume there are *K* candidate statistical models for a process of interest, one of them being the true underlying model. The models are indexed by the model indicator random variable $m \in \{1,2,\ldots ,K\}$. Each model *m* has a likelihood function $p(\mathbf {y}|\varvec{\theta }_m,m,\mathbf {d})$, with data $\mathbf {y}\in \mathscr {Y}$, and parameter vector $\varvec{\theta }_m \in \Theta _m$. In the experimental design context, the likelihood depends on the design vector $\mathbf {d}\in \mathscr {D}$, which is a vector of controllable variables of the experiment that might influence the informativeness of the data $\mathbf {y}$. In the Bayesian framework, a prior distribution $p(\varvec{\theta }_m|m)$ is assigned to the parameters of each model *m*. Furthermore, we assign a prior probability *p*(*m*) to each model such that $\sum _{m=1}^K p(m) = 1$. One can then derive the following important quantities from these elements: $p(\mathbf {y}|m,\mathbf {d}) = \int _{\varvec{\theta }_m} p(\mathbf {y}|\varvec{\theta }_m,m,\mathbf {d}) \, p(\varvec{\theta }_m|m) \, \mathrm {d} \varvec{\theta }_m$ is the *marginal likelihood*, *evidence*, or *prior predictive distribution* for model *m*; $p(\mathbf {y}|\mathbf {d}) = \sum _{m=1}^K p(\mathbf {y}|m,\mathbf {d}) \, p(m)$ is the overall or model-averaged *marginal likelihood* or *prior predictive distribution*; and $p(m|\mathbf {y},\mathbf {d}) = p(\mathbf {y}|m,\mathbf {d}) \, p(m) \bigl / p(\mathbf {y}|\mathbf {d})$ is the *posterior model probability* of model *m*.

Optimal experimental design requires the specification of a design criterion that encodes the goal of the experiment. In Bayesian design, a function *l* that quantifies the loss of an experiment needs to be specified, see, e.g. Overstall et al. (2018). Apart from the design $\mathbf {d}$, this loss function usually also depends on the model indicator *m* and the data $\mathbf {y}$ observed at the experiment. It may also depend on the parameters $\varvec{\theta }_m$ at each of the models. For experimental design, the expected or integrated loss,

1 $$\begin{aligned} l(\mathbf {d}) = \mathrm {E}_{\varvec{\theta }_m,\mathbf {y},m|\mathbf {d}}[\, l(\mathbf {d},\varvec{\theta }_m,\mathbf {y},m)], \end{aligned}$$

is of interest, where the expectation is taken with respect to all the unknown variables. The optimal design is given by $\mathbf {d}^* = \arg \min _{\mathbf {d}\in \mathscr {D}}l(\mathbf {d})$, where $\mathscr {D}$ is the set of admissible designs, which in general is a challenging optimisation problem. Alternatively, the design problem may be formulated in terms of a utility function instead of a loss function. Then, the goal is to maximise the expected utility function.

In Bayesian model discrimination, we are interested in finding a design $\mathbf {d}$ that is likely to produce data $\mathbf {y}$ from which we can infer the posterior distribution of the model indicator *m* with minimal uncertainty. The most popular measure of uncertainty of a distribution is its *Shannon entropy* (see, e.g. Lindley 1956). For a given data set $\mathbf {y}$, the conditional entropy of the model indicator is given by

$$\begin{aligned} l_{MD}(\mathbf {d},\mathbf {y}) = - \sum _{m=1}^K p(m|\mathbf {y},\mathbf {d})\log p(m|\mathbf {y},\mathbf {d}). \end{aligned}$$

The conditional entropy features the loss function

$$\begin{aligned} l_{MD}(\mathbf {d},\mathbf {y},m) = - \log p(m|\mathbf {y},\mathbf {d}), \end{aligned}$$

which is called the *multinomial deviance loss* (Hastie et al. 2009).

Since $\mathbf {y}$ is not known in advance, we take the average over the marginal distribution of $\mathbf {y}$, $p(\mathbf {y}|\mathbf {d})$. For discrete data $\mathbf {y}$, the expected multinomial deviance loss is

2 $$\begin{aligned} l_{MD}(\mathbf {d}) = - \sum _{\mathbf {y}\in \mathscr {Y}} p(\mathbf {y}|\mathbf {d}) \sum _{m=1}^K p(m|\mathbf {y},\mathbf {d})\log p(m|\mathbf {y},\mathbf {d}). \end{aligned}$$

The negative of the expected multinomial deviance loss is also known as the *mutual information utility* (see, e.g. Drovandi et al. 2014).

Another common loss function for model discrimination is the *0–1 loss* (see, e.g. Overstall et al. 2018). Let $\hat{m}(\mathbf {y}|\mathbf {d})$ be a classifier function that assigns one of the class labels $1,\ldots ,K$ to the data $\mathbf {y}$. The 0–1 loss function is defined as

$$\begin{aligned} l_{01}(\mathbf {d},\mathbf {y},m) = \mathrm {I}[\hat{m}(\mathbf {y}|\mathbf {d}) \ne m] = 1 - \mathrm {I}[\hat{m}(\mathbf {y}|\mathbf {d}) = m], \end{aligned}$$

where $\mathrm {I}[\cdot ]$ is the indicator function, which takes the value 1 if the argument is true and 0 otherwise. Therefore, the 0–1 loss is 1 if the data are misclassified and 0 if they are classified correctly. A generalisation of this loss function would be a loss matrix that assigns different loss values to all the combinations of true and selected models. Averaging the 0–1 loss function over the prior predictive distribution of the data and the model indicators yields the *misclassification error rate* or *prior error rate* (Pudlo et al. 2016), which for discrete data $\mathbf {y}$ is given by

3 $$\begin{aligned} l_{01}(\mathbf {d}) = \sum _{\mathbf {y}\in \mathscr {Y}} p(\mathbf {y}|\mathbf {d}) \sum _{m=1}^K p(m|\mathbf {y},\mathbf {d}) \{1 - \mathrm {I}[\hat{m}(\mathbf {y}|\mathbf {d}) = m]\}.\nonumber \\ \end{aligned}$$

The classifier $\hat{m}(\mathbf {y}|\mathbf {d}) = \arg \max _{m \in \{1,\ldots ,K\}} p(m|\mathbf {y},\mathbf {d})$—also known as the *Bayes classifier*—classifies the data according to the posterior modal model. It can be shown that the Bayes classifier minimises the expected 0–1 loss (3). The misclassification error rate for the Bayes classifier is called the *Bayes error rate* (see Hastie et al. 2009).

In the continuous case, the sums over $\mathbf {y}\in \mathscr {Y}$ in the expected loss functions (2) and (3) have to be replaced by integrals. The integrals and sums involved in (2) and (3) can be high-dimensional, analytically intractable and computationally intensive to approximate accurately. One approach is to estimate the expected loss functions using Monte Carlo integration. Let $\mathbf {y}^{m,j} \sim p(\mathbf {y}|m,\mathbf {d})$ for $j=1,\ldots ,J_m$ and $m=1,\ldots ,K$. That is, $J_m$ draws $\mathbf {y}^{m,j}$ from the prior predictive distribution under model *m* are generated, for each of the models in turn. Then, we can estimate the expected loss (2) by

4 $$\begin{aligned} \hat{l}_{MD}(\mathbf {d}) = - \sum _{m=1}^K p(m) \frac{1}{J_m} \sum _{j=1}^{J_m} \log p(m|\mathbf {y}^{m,j},\mathbf {d}), \end{aligned}$$

and the expected loss (3) by

5 $$\begin{aligned} \hat{l}_{01}(\mathbf {d}) = 1 - \sum _{m=1}^K p(m) \frac{1}{J_m} \sum _{j=1}^{J_m} \mathrm {I}[\hat{m}(\mathbf {y}^{m,j}|\mathbf {d}) = m], \end{aligned}$$

respectively, where $$\hat{m}(\mathbf {y}^{m,j}|\mathbf {d}) = \arg \max _{m \in \{1,\ldots ,K\}} p(m|\mathbf {y}^{m,j},\mathbf {d})$$.

The first issue with these approximations is that $J_m$ may need to be large to estimate the expected loss with low variance. The second issue is that the posterior model probability, $p(m|\mathbf {y},\mathbf {d})$, is generally not available analytically and is difficult to approximate accurately. In fact, estimating this quantity is a research problem in its own right in the Bayesian community (Friel and Wyse 2012). For an efficient recent approach using Gaussian quadrature, see Chai et al. (2019). In the Bayesian optimal design setting, an estimate of the expected loss requires $J = \sum _{m=1}^K J_m$ evaluations/approximations of $p(m|\mathbf {y},\mathbf {d})$, one for each data set $\mathbf {y}$ drawn from the prior predictive distribution. Then, the expected loss must be optimised over a potentially large design space $\mathscr {D}$, and therefore often many thousands of posterior model probabilities must be calculated to arrive at an optimal design. This is why only relatively simple models and experimental settings have been considered in the Bayesian design literature for model discrimination in comparison with the elaborate models that can be analysed in Bayesian inference (see, e.g. the application in Drovandi et al. 2014).

Further complications arise for estimating $p(m|\mathbf {y},\mathbf {d})$ when the likelihood function $p(\mathbf {y}|\varvec{\theta }_m,m,\mathbf {d})$ for the models of interest is computationally intractable. Dehideniya et al. (2018b) present a rather general ABC approach to tackle the problem of Bayesian design for model discrimination for models with intractable likelihoods. However, their approach is very simulation-intensive and therefore only suitable for low-dimensional designs. The approach of Overstall and McGree (2019) relies on finding suitable auxiliary models for the intractable models of interest and uses Gaussian processes to model the relationship between the parameters of the true model and the corresponding auxiliary model parameters. The marginal likelihood is modelled by a copula, which aims to capture the dependence induced by marginalising out the parameters. Dehideniya et al. (2018a), on the other hand, use a synthetic likelihood approach to approximate the true likelihood function. This approach works best if the likelihood function depends on summary statistics whose distribution is close to normal. A more computationally efficient approach is presented in Dehideniya et al. (2019), where Laplace-based approximations are used to estimate the design criteria instead of performing Monte Carlo integration. In order to find the posterior mode and curvature required for the Laplace approximation, synthetic likelihoods are used.

The ultimate goal of this paper is to expand the set of models and design settings for which it is possible to obtain optimal Bayesian designs for the purpose of model discrimination without having to rely on the availability of suitable parametric likelihood approximations.

## The classification approach

### Methodology

In this paper, we take a classification perspective on the Bayesian model discrimination problem to greatly reduce the computational burden highlighted in the previous section. As a by-product, we also obtain several other advantages over the standard Bayesian approach. The only requirement to apply our methodology is that it is computationally efficient to simulate from each of the *K* models. Therefore, the class of models that can be considered in optimal design for model selection increases dramatically. In addition, the generality of the proposed approach allows for implementations that are less application-specific. Furthermore, we find that the performance of the optimal design can be assessed easily via the misclassification error rate, as opposed to performing more posterior calculations at the optimal and sub-optimal designs.

For each design $\mathbf {d}$ proposed in the design optimisation algorithm, our approach involves simulating *J* samples from the joint distribution of data and model indicators,

$$\begin{aligned} p(\mathbf {y},m|\mathbf {d}) = \int _{\varvec{\theta }_m} p(\mathbf {y}|\varvec{\theta }_m,m,\mathbf {d}) \, p(\varvec{\theta }_m|m) \, p(m) \, \mathrm {d} \varvec{\theta }_m, \end{aligned}$$

to generate the training sample $\mathscr {T}= \{ (m^j,\mathbf {y}^j): \, j = 1,\ldots ,J\}$.

We can use this training sample to train a supervised classification algorithm, where we consider the model indicator *m* as a categorical response or ‘target’ variable and the simulated data $\mathbf {y}$ as the features. As a result, we obtain a classifier function $\hat{m}_{C}(\mathbf {y}|\mathbf {d},\mathscr {T})$ that we can use in Equation (5) instead of the Bayes classifier to estimate the misclassification error rate.

Alternatively, we can write the sample $\mathscr {T}$ as

$$\begin{aligned} \mathscr {T}= \left\{ (m, \, \mathbf {y}^{m,j}): \, j=1,\ldots ,J_m; \, m = 1,\ldots ,K \right\} , \end{aligned}$$

where $J_m$ is the number of samples from model *m* in $\mathscr {T}$. Given *m*, the data are sampled from $\mathbf {y}^{m,j} \sim p(\mathbf {y}|m,\mathbf {d})$. The numbers $J_m$ may be fixed in advance, usually selected to be proportional to the prior model probabilities. However, if the prior model probabilities are highly imbalanced, there may only be a few observations from the models with small prior model probabilities in $\mathscr {T}$. For training the classifier, it may then be advantageous to have a more balanced training sample. If the sample proportions do not reflect the prior model probabilities, it is necessary to adjust the classifier accordingly, for example by weighting the observations.

Due to overfitting, it is not advisable to use the same sample $\mathscr {T}$ for training the classifier as well as for evaluating the expected 0–1 loss in (5). To deal with this problem, one possibility to estimate the expected loss in practice is to use *L*-fold cross-validation (see, e.g. Hastie et al. 2009), where the full sample $\mathscr {T}$ is randomly split into *L* folds of approximately equal size: $\mathscr {T}= \{\mathscr {T}^{1},\ldots ,\mathscr {T}^L\}$. Let $\mathscr {T}^{-i} = \{\mathscr {T}^{1},\ldots ,\mathscr {T}^{i-1},\mathscr {T}^{i+1},\ldots ,\mathscr {T}^L\}$ denote the full sample without the *i*th fold, and let $\mathscr {T}^i_m$ be defined as $\mathscr {T}^i_m = \{\mathbf {y}_*: (m_*,\mathbf {y}_*) \in \mathscr {T}^i \, \wedge \, m_*=m \}$. The procedure is repeated *L* times. At each step *i* ($i = 1,\ldots ,L$), the classifier is trained on $\mathscr {T}^{-i}$ and validated on the subsample $\mathscr {T}^i$. Thus, at step *i* the expected 0–1 loss is computed as

6 $$\begin{aligned} \hat{l}_{01,i}^{\mathrm {cv}}(\mathbf {d}) = 1 - \sum _{m=1}^K p(m) \frac{1}{J_m^i} \sum _{\mathbf {y}\in \mathscr {T}_m^i} \mathrm {I}[\hat{m}_{C}(\mathbf {y}|\mathbf {d},\mathscr {T}^{-i}) = m], \nonumber \\ \end{aligned}$$

where $$J_m^i = \mathrm {card}(\mathscr {T}_m^i)$$.

The final estimate of the expected 0–1 loss is then obtained by averaging over the *L* expected loss estimates:

7 $$\begin{aligned} \hat{l}_{01}^{\mathrm {cv}}(\mathbf {d}) = \frac{1}{L} \sum _{i=1}^L \hat{l}_{01,i}^{\mathrm {cv}}(\mathbf {d}). \end{aligned}$$

In our examples, we always perform stratified sampling of the fold indicators. That is, first we divide the total sample $\mathscr {T}$ into *m* subsamples according to the model indicators. Then, we randomly split the subsample for each model into *L* equal-sized folds. Finally, we combine all the subsample folds with the same fold indicator *i* across all model subsets into fold $\mathscr {T}^i$. In this way, we guarantee that the model proportions are the same in all folds.

An alternative to cross-validation would be to generate an independent test or validation sample and evaluate the expected loss function on that sample. Depending on how cheap it is to simulate the data and how expensive it is to run the classifier, this approach might be preferable to cross-validation. In our examples, we only report the results for cross-validation since both approaches are qualitatively very similar.

Larger values of *J* allow for a more accurate estimate of the misclassification error rate and therefore lead to a less noisy objective function to optimise over, although the time to estimate the error rate increases. However, for intractable likelihood models the sample size *J* needed for the classification approach to obtain a reasonably precise approximation of the expected loss function is several orders of magnitude less than the sample size required for ABC (Pudlo et al. 2016). Moreover, for many other models the classification approach may be more time-efficient than estimating $p(m|\mathbf {y},\mathbf {d})$ in a conventional way.

Many classification methods also provide estimates of the posterior model probabilities, $\hat{p}_C(m|\mathbf {y},\mathbf {d},\mathscr {T})$, which can be used to estimate the expected multinomial deviance loss (2) in a similar way as the misclassification error rate is estimated by Equations (6) and (7). However, the estimates for the posterior model probabilities provided by many computationally efficient methods such as classification trees or linear discriminant analysis are rather crude, noisy, and biased (see, e.g. Breiman et al. 1984; Hastie et al. 2009).

Even if the posterior model probabilities are estimated poorly, the classification method can perform quite well at the task of assigning the correct class labels to the observations. All that matters is that the posterior modal model is identified correctly. If a classifier assigns the posterior modal model $\arg \max _m p(m|\mathbf {y},\mathbf {d})$ to each data set $\mathbf {y}\in \mathscr {Y}$, it is called an *order-correct classifier* (Breiman 1996). For an order-correct classifier, the misclassification error rate corresponds to the Bayes error rate and is therefore minimal. The misclassification error rate of a classifier that is order-correct everywhere except for a small subset of the sample space $\mathscr {Y}$ will still be very close to the Bayes error rate. Therefore, the misclassification error rate is relatively robust to inaccurate estimates of the posterior model probabilities. For this reason, we focus mainly on finding designs which are optimal with respect to the misclassification error rate. However, the misclassification error rate is not estimated very well if the posterior modal model is hard to identify among several highly probable models in a non-negligible subset of the sample space $\mathscr {Y}$, which may happen, for example, if the data are generally not very informative.

### CARTs and random forests

There are a plethora of supervised classification algorithms that are suitable candidates for the task of estimating the expected loss. As the optimal design procedure estimates the expected loss many times, we require a fast classification method. As a generic and fast nonparametric classification approach, we adopt *classification and regression trees* (CART, see Breiman et al. 1984) to estimate the expected loss at each design visited during the design procedure.

One disadvantage of trees is their high variance. Slight changes in the data might lead to widely different trees. To reduce the variance, Breiman (2001) proposes *random forests*, which consist of an ensemble of trees. For classification, the class prediction of a random forest is obtained by majority vote among the individual trees of the forest. More information about the structure, properties, and estimation of CARTs and random forests can be found in Online Resource 1 (Sect. 1).

Random forests have been used successfully in many applications and compare favourable to/with many other more computationally intensive classification methods such as boosting or neural networks; see Hastie et al. (2009). Their nonparametric nature allows for capturing complex dependencies between the model indicator and the features, and so they are more flexible than many parametric methods such as logistic regression. Another advantage of trees and random forests is that the scaling of the features does not matter, so there is no need to standardise or transform the features. For our purpose, it is also important that random forests do not require any tuning for each new data set and design because the standard settings work reasonably well in most situations. A further advantage of random forests is that the misclassification error rate can be estimated using *out-of-bag* class predictions (Breiman 2001), so there is no need to perform cross-validation or to generate a test set.

Pudlo et al. (2016) note that random forests can easily cope with many noisy, weakly informative and correlated input features. Nevertheless, if the dimension of the raw data is very high, summary statistics may need to be used to improve the classification performance. However, random forests make it possible to include a relatively large amount of informative summary statistics. This may alleviate the loss of information regarding model discrimination when using non-sufficient summary statistics reported by Robert et al. (2011). The standard kernel-based ABC approaches for intractable likelihood problems suffer from the curse of dimensionality much more strongly and require low-dimensional summary statistics to work efficiently (see, e.g. Blum 2010).

It is possible to obtain estimates for the posterior model probabilities $p(m|\mathbf {y},\mathbf {d})$ from trees and random forests. However, these estimates are not smooth and very rough, in particular for trees. It might happen that the estimated posterior model probabilities for some observations are 0, which causes problems when estimating the expected multinomial deviance loss. Section 1 of Online Resource 1 discusses this issue in more detail and explains how we deal with it.

### Assessing the performance of a design

Once we have found optimal or close-to-optimal designs using a variety of our design search methods for some design dimensions, we are also able to assess the performance of those designs with the classification method. For example, it may be of interest to assess the ability to discriminate between models as the sample size or design dimension is increased, or to investigate which design search methods lead to more efficient designs. We want this assessment to be as accurate as possible. Given that only a relatively small number of designs need to be assessed, we suggest that more effort can be placed in the classification procedure. For example, we can simulate both a large training and a large test set and fit an elaborate classifier such as a random forest with a large number of trees. Then, the classification performance in terms of the misclassification error rate can be estimated by applying the fitted model to the test data set.

## Examples

In this section, we consider several examples to highlight the utility of our proposed method. To perform the design optimisation, we use a modification of the coordinate exchange (CE) algorithm (Meyer and Nachtsheim 1995), which involves cycling through each of the design variables iteratively, trialling a set of candidate replacements and updating the value of the design variable if the objective/loss function is reduced. This is continued until no updates to the design are made in a given cycle. To guard against possible local optima, we run the algorithm in parallel 20 times with random starts. We acknowledge the stochastic nature of our objective function by considering the (up to) six last designs visited in each of the 20 runs as candidates for the overall optimal design. For each of the candidates, we compute the loss function ten times to reduce the noise. The best design found through this algorithm is the one with the lowest average loss among the candidate designs across all runs. As an additional post-processing step, we combine all the candidate designs and estimated loss function values from all the runs. Then, we employ Gaussian process regression (Rasmussen and Williams 2006) on them to obtain a smooth estimate for the expected loss surface, which we seek to minimise with respect to the design. Finally, we compare the expected loss at this new design to the expected loss found previously by the coordinate exchange algorithm. This is done by estimating the expected loss 100 times at each of the two designs and selecting the design with the lower average expected loss as the optimal design. A detailed description of the optimisation algorithm that we employ is provided in Sect. 2 of Online Resource 1. We do not expend any effort on finding the best optimisation algorithm for each of the examples as this is not the focus of the paper. We find that the CE algorithm performs adequately to illustrate the findings of the paper.

The first example in Sect. 4.1 compares the results of our supervised classification approach to ABC for different loss functions for an infectious disease application. It demonstrates that ABC and the computationally much more tractable classification approaches lead to designs with similar efficiency. The second example in Sect. 4.2 is a modification of the first example. It only considers the first two models of the first example, which have reasonably tractable likelihoods. This makes it possible to obtain likelihood-based loss estimates and find likelihood-based designs at least for lower dimensions, which we can use for comparisons with our classification approach. In addition, we demonstrate how we are also able to apply our approach successfully to higher-dimensional design settings. The third example is a practically important application in the field of experimental biology. The goal is to obtain good designs for discriminating between different hypotheses about unobserved heterogeneity with respect to the reproduction of bacteria within phagocytic cells. We apply our classification-based design method to two further examples in Sects. 7 and 8 of Online Resource 1. The first is a fairly high-dimensional logistic regression example with fixed and random effects, for which previous attempts on finding Bayesian optimal designs were only possible by making some additional approximations (Overstall et al. 2018). The second example is an application to intractable max-stable spatial extremes models, for which designs were previously only found on a very limited number of candidate design points using the ABC approach (Hainy et al. 2016).

Listings of computational runtime performance statistics for the different methods and design settings for all the examples in this section can be found in Sect. 3 of Online Resource 1.

### Stochastic models in epidemiology

#### Problem formulation

An example involving four competing continuous-time Markov process models for the spread of an infectious disease is considered in Dehideniya et al. (2018b). Let *S*(*t*), *E*(*t*), and *I*(*t*) denote the number of susceptible, exposed, and infected individuals at time *t* in a closed population of size $N=50$ such that $S(t) + E(t) + I(t) = N$ for all *t*. The possible transitions in an infinitesimal time $\delta _t$ for each of the four models are shown in Table 1. Models 1–4 are referred to as the death, SI, SEI, and SEI2 models, respectively. Models 1 and 2 do not have an exposed population. The algorithm of Gillespie (1977) can be used to efficiently generate samples from all the models. The prior distributions for all the parameters of each model are provided in Table 5 of Online Resource 1. All models are assumed equally likely *a priori*.

**Table 1:** Four competing models considered in the infectious disease example of Section 4.1

Model	Event type	Update	Rate
1	Infected	$S(t)-1$, $I(t)+1$	$b_1^{(1)}S(t)$
2	Infected	$S(t)-1$, $I(t)+1$	$[b_1^{(2)} + b_2^{(2)}I(t)] \, S(t)$
3	Exposed	$S(t)-1$, $E(t)+1$	$b_1^{(3)}S(t)$
	Infected	$E(t)-1$, $I(t)+1$	$\gamma ^{(3)}E(t)$
4	Exposed	$S(t)-1$, $E(t)+1$	$[b_1^{(4)} + b_2^{(4)}I(t)] \, S(t)$
	Infected	$E(t)-1$, $I(t)+1$	$\gamma ^{(4)}E(t)$

We consider the design problem of determining the optimal times (in days) $\mathbf {d}= (d_1,d_2,\ldots ,d_n)$, where $d_1< d_2< \cdots < d_n \le 10$, to observe the stochastic process in order to best discriminate between the four models under the available prior information. Only the infected population can be observed. Unfortunately, the likelihood functions for all but the simplest model are computationally cumbersome as they require computing the matrix exponential (see, e.g. Drovandi and Pettitt 2008). Whilst computing a single posterior distribution is feasible, as in a typical data analysis, computing the posterior distribution or posterior model probabilities for thousands of prior predictive simulations, as in a standard optimal Bayesian design approach, is computationally intractable.

#### Approximate Bayesian computation

Dehideniya et al. (2018b) develop a likelihood-free approach based on approximate Bayesian computation (ABC) to solve this model discrimination design problem. Given a particular level of discretisation of the design space (time in this case), the ABC approach involves generating a large number of prior predictive simulations at all discrete time points and storing them in the so-called reference table. Then, for a particular ‘outer’ draw from the prior predictive distribution, $\mathbf {y}$, at some proposed design, $\mathbf {d}$, the ABC rejection algorithm of Grelaud et al. (2009) is used to estimate the posterior model probabilities and in further consequence the loss functions. This means that the posterior model probability $p(m|\mathbf {y},\mathbf {d})$ is estimated by computing the proportion of model *m* simulations in the retained sample, where the retained sample is composed of those simulations from the reference table which are ‘closest’ to the process realisation $\mathbf {y}$ with respect to some distance such as Euclidean or Manhattan distance. The size of the retained sample is only a very small fraction of the size of the reference table. The estimated posterior model probability is used to compute the estimated loss for process realisation $\mathbf {y}$. Finally, the estimated expected loss is obtained by averaging the loss estimates for all the ‘outer’ draws. The reader is referred to Dehideniya et al. (2018b) for more details. Price et al. (2016) improve the efficiency for these models by making use of the discrete nature of the data to efficiently estimate the expected loss.

#### Simulation settings

For each of the classification methods from machine learning, we use a sample of 5K simulations from each model to train the classifier and to estimate the expected loss at each new design. For the classification trees, we use tenfold cross-validation to estimate the expected loss functions. When using random forests, we employ out-of-bag class predictions. As a criterion, we use expected 0–1 loss as well as expected multinomial deviance loss. When computing the expected multinomial deviance loss, we set the posterior model probability of the correct model to 0.001 whenever it is estimated to be 0, see Sect. 1 of Online Resource 1 for more information. We could follow the ABC method and draw the simulations from a large bank of prior predictive process realisations simulated at the whole design grid to reduce the computing time. However, since the machine learning classification method requires significantly fewer simulations, we find that it is still fast to draw a fresh process realisation for each proposed design. For the ABC approach, the reference table contains 100K stored prior predictive simulations for each model. To compute the expected loss, we average the estimated loss over 500 ‘outer’ draws from $p(\mathbf {y}|m,\mathbf {d})$ for each model and retain a sample of size 2K from the reference table for each draw. For all the methods, the optimal design search was conducted over a grid of time points from 0.25 to 10 with a spacing of 0.25.

#### One-dimensional estimated expected loss curves

**Fig. 1:**
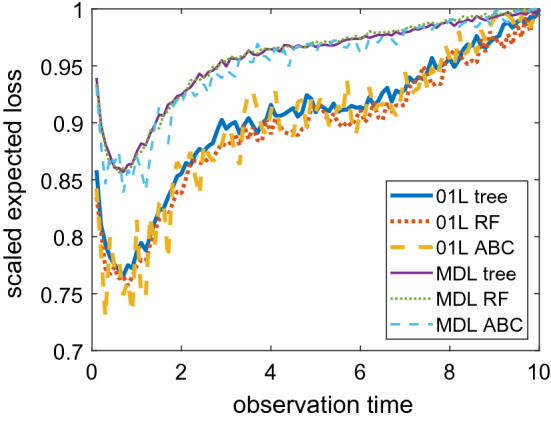
Plots of the approximated expected loss functions produced by the tree classification approach with cross-validation (solid), the random forest classification approach using out-of-bag class predictions (dotted), and the ABC approach (dashed) under the 0–1 loss (thick lines) and multinomial deviance loss (thin lines) for the infectious disease example. The expected losses have been scaled by dividing through the maximum loss for an easier comparison

Figure 1 shows the approximate expected loss functions for 1 design observation under several estimation approaches and loss functions over a grid of design points with spacing 0.1. It is evident that all the functions are qualitatively similar and produce the same optimal design around $0.5 - 0.7$ days. In particular, one can see that the expected loss curves for both the 0–1 loss and the multinomial deviance loss seem to be minimised at around the same observation time. However, the times needed to construct the curves are vastly different between the different approaches. On our workstation, it took less than half a minute for the cross-validated tree classification approach (single core), between 4 and 5 minutes for the random forest classification approach (single core), and between 9.5 and 10 minutes using 8 parallel cores for the ABC approach to generate the respective graphs. Creating the reference table with 400K simulations required only between 3.5 and 4 seconds in this example, since sampling via the Gillespie algorithm is very efficient. In our example, what is causing the computational inefficiency of ABC is having to sort the large reference table for each outer draw to obtain the retained ABC sample. Despite the much higher computational effort needed for the ABC approach, its estimates of the expected loss functions are still considerably noisier than the estimates of the classification approaches, which is mostly due to the relatively small outer sample size of 2000.

#### Optimal designs

The optimal designs obtained by the machine learning and ABC approaches are shown in Table 2 for $n=1$ to $n=3$ time points and Table 6 of Online Resource 1 for $n=4$ and $n=5$ time points. The machine learning methods lead to designs with a general preference for later sampling times. The designs obtained by trees and random forests are very similar. The ABC approach produces designs with notably lower sampling times. However, the results obtained by the ABC approach should be taken with caution, since the high noise of the expected loss estimates makes it harder to optimise over the design space, especially for higher dimensions. Moreover, the approximation of the posterior gets worse the higher the dimension. It is also interesting to note that there are hardly any differences between the two loss functions for any given method. This reaffirms our decision to consider only the 0–1 loss in the other examples.

**Table 2:** Optimal designs obtained by tree classification (cross-validated), random forest classification (using out-of-bag class predictions), and ABC approaches under the 0–1 loss (01L) or multinomial deviance loss (MDL) ($n = 1$, 2, and 3) for the infectious disease example. The equidistant designs are also shown

Method/Loss	$n = 1$	$n = 2$		$n = 3$		
Tree 01L	0.598	0.787	4.437	0.818	4.568	9.493
RF 01L	0.611	0.823	4.433	0.750	4.000	10.000
ABC 01L	0.597	0.877	4.357	0.750	2.250	5.750
Tree MDL	0.621	0.750	4.750	0.750	4.750	10.000
RF MDL	0.633	0.750	4.750	0.750	4.500	9.000
ABC MDL	0.556	0.750	3.500	0.500	1.750	4.750
Equidistant	5.000	3.333	6.667	2.500	5.000	7.500

#### Classification performance evaluations of optimal designs

As our next step, we compare the optimal designs found under the different approaches using a random forest classifier. For each of the optimal designs, we train a random forest with 100 trees based on 10K simulations from each model. The misclassification error rates and the misclassification matrices are estimated from a fresh set of 10K simulations from each model. This is repeated 100 times to be able to quantify the random error in estimating the misclassification error rates. The results for all the optimal designs as well as for the equispaced designs are shown in Table 3. For more than two observations, the designs that clearly perform best are those found under the machine learning classification approaches. However, also the ABC optimal designs generally perform well except for $n = 5$ design times. We can also observe that the loss function used for optimisation has little effect on the performance of the optimal design, only for the designs found using ABC there is a notable difference for $n = 4$. The equispaced designs perform substantially worse than all the optimal designs up until $n = 4$ observations. Table 3 also shows that there is almost no gain in the classification performance by increasing the number of observations beyond 2. Any additional observation will only add a negligible amount of information regarding model discrimination. At some point, adding additional uninformative observations adversely affects the classification power of the random forest.

**Table 3:** Average misclassification error rates for optimal designs obtained by tree classification (cross-validated), random forest classification (using out-of-bag class predictions), and ABC approaches under the 0–1 loss (01L) or multinomial deviance loss (MDL) as well as for the equidistant designs for the infectious disease example. The average misclassification error rates were calculated by repeating the random forest classification procedure 100 times (see text) and taking the average. The standard deviations are given in parentheses

Design	$n = 1$	$n = 2$	$n = 3$	$n = 4$	$n = 5$
Tree 01L	0.5554	0.5158	0.5133	0.5116	0.5129
	(0.0023)	(0.0024)	(0.0026)	(0.0022)	(0.0025)
RF 01L	0.5548	0.5160	0.5132	0.5113	0.5138
	(0.0027)	(0.0025)	(0.0025)	(0.0025)	(0.0025)
ABC 01L	0.5547	0.5161	0.5196	0.5046	0.5339
	(0.0023)	(0.0025)	(0.0030)	(0.0024)	(0.0027)
Tree MDL	0.5547	0.5178	0.5152	0.5183	0.5159
	(0.0023)	(0.0030)	(0.0028)	(0.0028)	(0.0025)
RF MDL	0.5550	0.5179	0.5118	0.5104	0.5128
	(0.0022)	(0.0026)	(0.0026)	(0.0026)	(0.0026)
ABC MDL	0.5553	0.5221	0.5216	0.5226	0.5416
	(0.0020)	(0.0028)	(0.0028)	(0.0029)	(0.0025)
Equidistant	0.6592	0.6200	0.5760	0.5537	0.5519
	(0.0029)	(0.0025)	(0.0027)	(0.0029)	(0.0032)

**Fig. 2:**
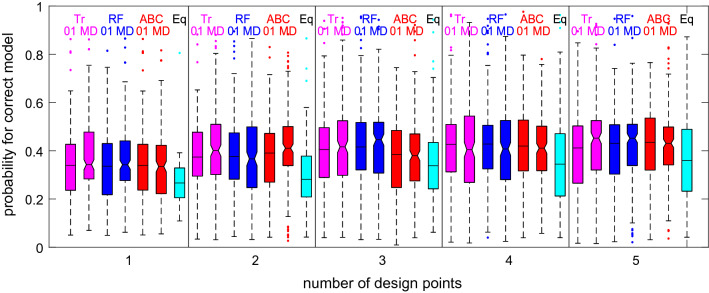
For each of the optimal designs obtained by the different approaches for 1–5 observations in the infectious disease example, display the distribution of estimated ABC posterior model probabilities of the correct model over 200 process realisations (50 from each of the four models) simulated from the prior predictive distribution at the respective optimal design. For each number of design points, from left to right there are two magenta box plots for the cross-validated tree classification designs, two blue box plots for the random forest classification designs, two red box plots for the ABC classification designs, and one cyan box plot for the equispaced design. Box plots for the 0–1 loss and for the equispaced designs do not have a notch, whereas box plots for the multinomial deviance loss are notched

Finally, we compare the optimal designs obtained by the different methods based on approximate posterior model probabilities estimated using ABC, as described in Sect. 4.1.2. To that end, for each design to evaluate we simulate 50 process realisations from the prior predictive distribution of each of the four models at that design and estimate the posterior model probability of the true model using ABC rejection. To get precise estimates of the posterior model probabilities for each of the 200 process realisations, we generate 10 million simulations from the prior predictive distribution to build the reference table. To estimate the posterior probabilities for each generated process realisation, we retain the 40K simulations from the reference table closest to that process realisation with respect to the Manhattan distance of the standardised observations. Box plots showing the distributions of the estimated model probabilities over the 200 prior predictive process realisations for all the optimal designs as well as for the equispaced designs for 1–5 observations are plotted in Fig. 2. It can be seen that the results for all the different optimal designs are very similar, even though the approaches using the 0–1 loss criterion do not directly target the improvement in the posterior model probabilities. The equispaced designs perform appreciably worse up until $n = 4$ observations. It is also evident that, given the prior information in this example, not much gain can be achieved by collecting more than two observations, which is similar to the random forest classification results obtained in Table 3. Assessing the optimal designs using random forests is much faster than performing this ABC simulation study.

A more detailed investigation of the classification performance at the optimal designs can be found in Sect. 4.3 of Online Resource 1.

### Two-model epidemiological models with true likelihood validation

#### Aims and model set-up

For the example in this section, we use the same infectious disease model set-up as in Sect. 4.1. However, only the death and SI models (models 1 and 2) from Table 1 are considered. The reason is that for these two models the computation of the likelihood function is efficient enough to be able to compute likelihood-based posterior model probabilities for a sufficiently large amount of prior predictive samples. Therefore, we can compare the results for our likelihood-free approach using supervised classification methods to the results obtained by using the true likelihood functions to estimate the design criterion. Furthermore, we can assess the resulting optimal designs by computing the expected posterior model probabilities and misclassification error rates based on the true likelihood functions.

Another aim of this example is to demonstrate that the classification approach can easily cope with higher-dimensional designs, where other methods would fail to produce reasonable results in an acceptable amount of time. For the epidemiological example with four models in Sect. 4.1, one can see that there is hardly any gain in increasing the number of design points beyond three, so it makes no sense to consider any higher-dimensional designs. However, in Sect. 4.1 we assume that we can only observe one realisation of the infectious disease process. In this section, in order to explore the performance of our methods for high-dimensional designs, we assume that several independent realisations of the stochastic process can be observed. For example, these independent realisations may pertain to independent populations of individuals. We allow each realisation to be observed at potentially different time points.

For simplicity, we assume that the same number of observations, $n_d$, is collected for each realisation. If there are *q* realisations, then the total number of observations and therefore the design dimension is $n = q \cdot n_d$.

The prior distributions for the parameters are $b_1 \sim {\mathscr {L}}{\mathscr {N}}(\mu = -0.48, \sigma = 0.3)$ for the death model and $b_1 \sim \mathscr {LN}(\mu = -1.1, \sigma = 0.4)$, $b_2 \sim \mathscr {LN}(\mu = -4.5, \sigma = \sqrt{0.4})$ for the SI model.

In this example, we will only consider designs based on using the misclassification error rate as the design criterion. In order to compute the misclassification error rates based on the likelihoods, it is necessary to compute the marginal likelihoods for both models. When searching for the optimum design, we employ a relatively fast Laplace-type approximation to the marginal likelihood. For validating the resulting designs using the likelihood-based approach, we use a more expensive Gauss–Hermite quadrature scheme to obtain the marginal likelihoods. Details on both integral approximation methods can be found in Sect. 5.2 of Online Resource 1.

#### Example settings and results

When searching for the optimal designs, we employ trees with cross-validation as well as random forests using out-of-bag class predictions for our supervised classification approach. For both classification approaches, we use simulated samples of size 10K (5K per model).

**Fig. 3:**
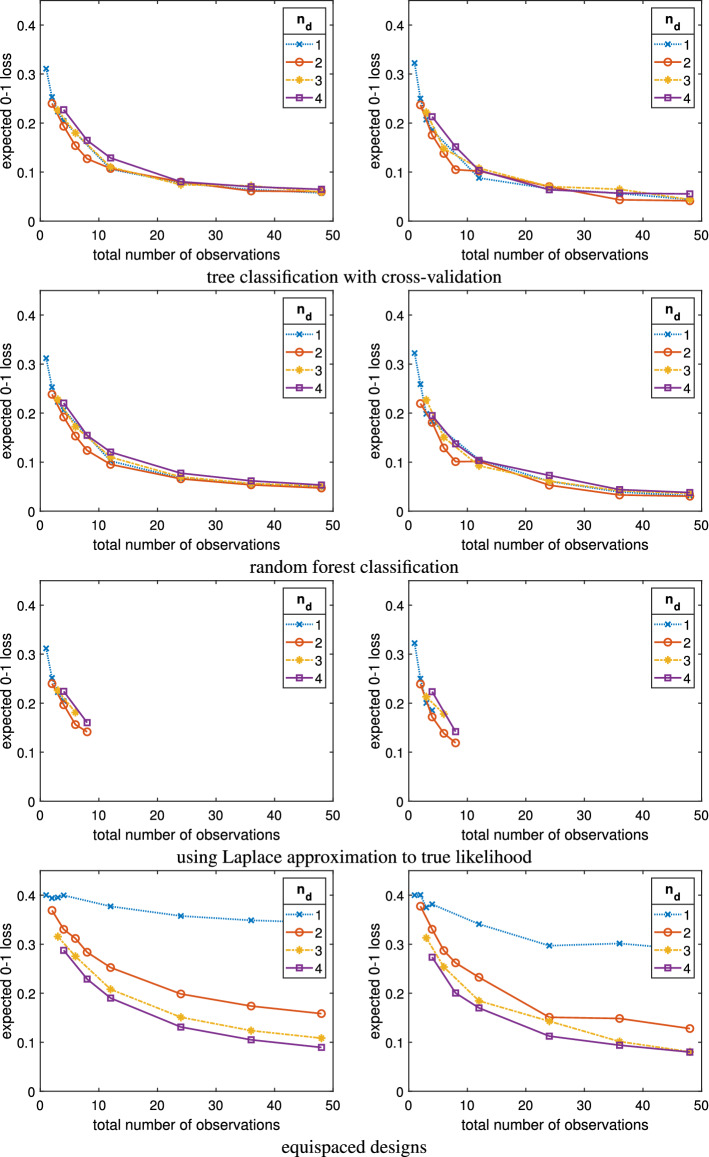
Misclassification error rates computed using random forest classification with training and test samples of size 20K, averaged over 100 repetitions of the classification procedure (left column) and misclassification error rates computed using the Gauss–Hermite quadrature approximation to the marginal likelihood over 2K prior predictive simulations (right column) evaluated at various optimal designs for different methods (in the rows) for the infectious disease example with two models. The total number of observations ($n = q \cdot n_d$) is plotted on the x-axis of each graph. Each line connects the observed values of the misclassification error rate as the number of realisations *q* increases for a particular value of $n_d$

For the likelihood-based approach, the expected 0–1 loss (= misclassification error rate) is estimated by averaging the computed 0–1 loss over a sample of size 400 (200 per model) from the prior predictive distribution. The size of this prior predictive sample is considerably smaller than for the two supervised classification approaches due to computational limitations. Therefore, the volatility of our likelihood-based misclassification error rate estimates is much higher than for the supervised classification methods, so we expect our optimisation procedure to be less stable. The expected loss surface for the one-dimensional design is depicted in Fig. 3 of Online Resource 1.

However, setting the prior predictive sample size for the likelihood-based approach to 10K as well would have made it infeasible to find an optimal design in a reasonable amount of time. Running the classification methods is still much more time-efficient than evaluating the likelihood function many times, especially for the SI model in high dimensions, see also Sect. 3 of Online Resource 1. Therefore, we only used the likelihood-based approach to find designs up to a total design dimension of $n = 8$. Furthermore, for the design search we used a relatively coarse grid with a spacing of 0.5 days between the limits 0.5 and 10 days. We used the same design grid for all approaches.

We consider various combinations of the number of realisations, *q*, and the number of observations per realisation, $n_d$. All the design methods described in this section are applied to all integer combinations of $1 \le n_d \le 4$ and $1 \le q \le 4$ for which the total number of observations $n = q \cdot n_d$ does not exceed 8. We also investigate higher-dimensional designs, where we only employ the supervised classification approaches but not the likelihood-based approach. As higher-dimensional settings we consider all integer combinations of *q* and $n_d$ which amount to a total number of observations of either $n = 12$, 24, 36, or 48, and where $1 \le n_d \le 4$.

The optimal designs found with the different methods are validated in two ways. Firstly, for each observation from a sample of size 2K (1K per model) from the prior predictive distribution, the posterior model probabilities are computed using the generalised Gauss–Hermite quadrature approximation to the marginal likelihood with $Q=30$ quadrature points for the death model and up to $Q=30^2$ quadrature points (minus some pruned points) for the SI model. The resulting distributions of posterior model probabilities are displayed in Section 5.3 of Online Resource 1.

We can also use the estimates for the posterior model probabilities to compute estimates of the misclassification error rates for each of the methods and dimension settings. These estimates are provided in the plots on the right-hand side of Figure 3, where each row contains the results for one design method, the x-axis of each plot shows the total number of observations, *n*, and each line within each plot displays the results for a particular setting of $n_d$. Alternatively, one can use a supervised classification method to estimate the misclassification error rates for the optimal designs. In our case, we use a random forest with training and test sets of size 20K (10K per model). The random forest classification procedure is repeated 100 times, and the average misclassification error rate over the 100 repetitions is taken. The random forest-based validation results are shown in the plots on the left-hand side of Fig. 3 analogous to the likelihood-based validation results.

From Figure 3, it is evident that the misclassification error rates computed by the random forests are very close to the likelihood-based misclassification error rate computations. In most cases, the random forest-based estimates of the misclassification error rate are a little higher than the likelihood-based estimates. This is no surprise since the likelihood-based estimates are directly targeting the Bayes error rate. However, the trajectories of the misclassification error rates as a function of *n* are very similar for both validation methods. This suggests that for this example random forests are suitable to validate and compare the efficiency of the resulting designs. In addition, it is reasonable to expect that the designs which are optimal for the random forest classification approach are close to the true optimal designs.

One can also see from Fig. 3 that for a fixed total number of observations there is not much difference in the performance of the different design configurations, at least for the small values of $n_d$ that we considered. It seems that having $n_d = 2$ observations per realisation is the most optimal choice, but only by a small margin.

### Macrophage model

#### Aim of experiment

A common challenge in experimental biology is identifying the unobserved heterogeneity in a system. Consider for example the experimental system in Restif et al. (2012). In this system, the authors wished to identify the role of antibodies in modulating the interaction of intracellular bacteria—in particular, *Salmonella enterica* serovar Typhimurium (*S.* Typhimurium)—with human phagocytes, inside which they can replicate. The experiments assessed the effect of a number of different human immunoglobulin subclasses on the intracellular dynamics of infection by combining observed numbers of bacteria per phagocyte with a mathematical model representing a range of different plausible scenarios. These models were fit to experimental data corresponding to each human immunoglobulin subclass in order to determine the underlying nature of the interactions between the antibodies and bacteria. In these experiments, the data demonstrated bimodal distributions in the number of intracellular bacteria per phagocytic cell. The aim was to identify the source of the unobserved heterogeneity in the system that caused the observed patterns. Specifically, is there underlying heterogeneity in the bacteria’s ability to divide inside phagocytes, or is it the phagocyte population which is heterogeneous in its ability to control bacterial division? In this context, the classification approach allows us to find the experimental design which best enables us to discriminate between these competing hypotheses—(1) unobserved heterogeneity in the bacteria, (2) in the cells, or (3) no heterogeneity.

#### Experimental procedure

We give a brief account of the experimental procedure:

- After bacterial opsonisation (i.e. the process by which bacteria are coated by antibodies), the bacteria are exposed to the phagocytic cells for a total of $t_{exp}$ hours, which can take the values $t_{exp} \in \{0.10, 0.20, \ldots , 1.50\}$ hours. During this time, phagocytosis occurs, i.e. the bacteria are internalised by the phagocytic cells.
- Next, the cells are treated with gentamicin, an antibiotic that kills extracellular bacteria, so that phagocytosis stops.
- At each of the *n* observation times $\varvec{t}_{obs}=(t_1,\dots ,t_n)$ hours post-exposure, two random samples of *S* cells each are taken from the overall population of cells: one sample to count the proportion of infected cells (under a low-magnification microscope), and one sample of infected cells to determine the distribution of bacterial counts per infected cell (at higher magnification).

That is, a design is composed of $\mathbf {d}=(t_{exp}; \varvec{t}_{obs})$. The full experimental procedure is detailed in Restif et al. (2012).

For the purpose of our example, we consider a realistic scenario where we have the resources to count a fixed number of cells, $N_{cells}=200$. These cells are then equally split between all the observation times and the two independent observational goals at each observation time, so $S = \lfloor N_{cells} / (2 \, n) \rfloor$.

#### Model

We consider three mathematical models, based on Restif et al. (2012), to represent the three competing hypotheses about heterogeneity. These models are continuous-time Markovian processes that simulate the dynamics of intracellular bacteria within macrophages. Model (1) tracks the joint probability distribution of the number of replicating and non-replicating bacteria within a single macrophage, assuming all macrophages in a given experiment are from the same type. In model (2), each macrophage has a fixed probability *q* of being refractory, in which case it only contains non-replicating bacteria, and a probability $1-q$ of being permissive, in which case it only contains replicating bacteria. In model (3), all macrophages are permissive and all bacteria are replicating.

Simulations from the models are based on simulations of bacterial counts for the individual macrophages. As for our infectious disease examples, we can use the efficient Gillespie algorithm (Gillespie 1977). The outcomes for the individual macrophages are then aggregated to obtain the same type of data as observed in the real experiment.

It is possible but cumbersome to compute the likelihood functions for all the models. Computing the likelihood involves solving a system of linear differential equations, which can be achieved by using matrix exponentials. However, these operations are quite expensive so that computing the posterior model probabilities becomes very costly. Computing the expected losses and searching for an optimal design can be considered intractable in these circumstances. In contrast, simulations from the models can be obtained very quickly.

Section 6.1 of Online Resource 1 contains a more detailed description of the Markov process models, the simulation procedure, the likelihood function, and the prior distributions.

#### Results

We use the machine learning classification approach using classification trees with cross-validation or random forests to determine the optimal designs for discriminating between the three competing models (one model corresponding to each hypothesis) with respect to the misclassification error rate. It is assumed *a priori* that the models are equally likely. We use 5K simulations from the prior predictive distribution of each model during the design process. The design grid for $t_{obs}$ goes from 0.25 to 10 with a spacing of 0.25. The optimal designs are given in Section 6.2 of Online Resource 1. The tree and the random forest classification approaches lead to very similar designs.

Similar to the other examples, we assess each design by producing 10K new simulations under each model at that design and using these to train a random forest with 100 trees. A further 10K new simulations per model are then used to estimate the misclassification error rate. This is repeated 100 times for each design. The estimated misclassification error rates for the designs found under the tree and random forest classification approaches are shown in Table 4. For comparison, we also include the estimated error rates for the equispaced designs.

**Table 4:** Average misclassification error rates for the optimal designs obtained under the classification approaches using trees or random forests and for the equispaced designs for the macrophage model. The average misclassification error rates were calculated by repeating the random forest classification procedure 100 times and taking the average. The standard deviations are given in parentheses

Design	$n = 1$	$n = 2$	$n = 3$	$n = 4$	$n = 5$
Tree	0.1928	0.1323	0.1433	0.1469	0.1483
	(0.0024)	(0.0021)	(0.0022)	(0.0019)	(0.0022)
RF	0.1925	0.1325	0.1408	0.1410	0.1465
	(0.0022)	(0.0021)	(0.0022)	(0.0021)	(0.0021)
Equi	0.2442	0.1974	0.1928	0.1912	0.1935
	(0.0027)	(0.0023)	(0.0023)	(0.0025)	(0.0024)

**Fig. 4:**
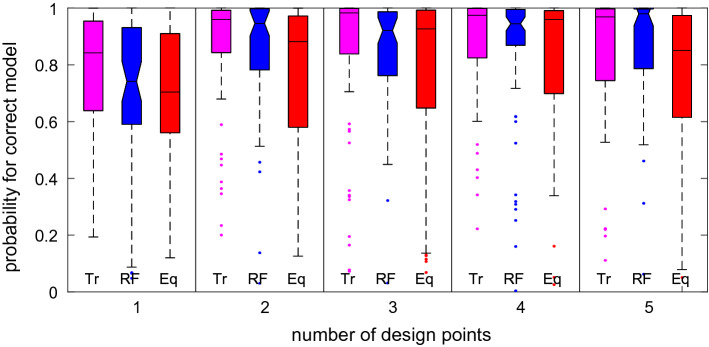
For each of the optimal designs obtained by the different approaches for 1–5 observations in the macrophage example, display the distribution of estimated posterior model probabilities of the correct model over 80 process realisations (20 from each of the three models) simulated from the prior predictive distribution at the respective optimal design. For each number of design points, the magenta box plot on the left-hand side is for the tree classification design, the notched blue box plot in the middle is for the random forest classification design (rf), and the red box plot on the right-hand side is for the equispaced design

We are also interested in the posterior model probabilities at the different optimal designs. At each optimal design, we simulate 20 process realisations under the prior predictive distribution of each model. For each process realisation, we approximate the posterior model probability of the model that generated the data using importance sampling (see, e.g. Liu 2001) with 50K simulations from the importance distribution. In our case, the prior distribution serves as the importance distribution. Figure 4 shows box plots for the distributions of the posterior model probabilities of the correct model over the prior predictive simulations for different optimal designs. The computations required to generate one of these box plots ranged from 5.9 hours to 17.2 hours using up to 24 parallel threads. In contrast, it took less than two minutes to obtain one estimate of the misclassification error rate using a random forest with training and test samples of size 30K each.

Table 4 indicates that $n = 2$ observation times yield the optimal classification power when using trees and random forests, even though the posterior model probabilities of the correct model keep increasing until at least $n = 4$ (see Fig. 4). For more than two observations, the higher data dimension impedes the classification accuracy of those classification methods and more than offsets the gains from having marginally more information in the data due to the more optimal allocation of resources to the different observation times. However, there are no substantial improvements in the posterior probabilities after $n = 2$. Both machine learning classification approaches lead to very efficient designs for all design sizes.

Overall, the ability to correctly classify output from the three models and thus to decide between the three competing hypotheses is very good at all the optimal designs. This suggests that we are able to identify with high certainty if heterogeneity is present, and if so, whether the bacteria or the human cells are the source of this heterogeneity.

## Discussion

We introduce a new simulation-based Bayesian experimental design approach for model discrimination where the expected loss is estimated via a supervised classification procedure. This approach requires significantly fewer simulations than other simulation-based approaches based on ABC. Furthermore, efficient, flexible, and fast classification methods such as classification trees or random forests can cope with medium to high data dimensions without imposing strict structural assumptions. Therefore, the classification approach significantly increases the scope of design problems which can be tackled compared to previous approaches. For example, optimal designs for the hierarchical logistic regression example could previously only be obtained by assuming normal-based approximations (Overstall et al. 2018). The high dimensions of the summary statistics for the macrophage and the spatial extremes example render the ABC approach unsuitable or even infeasible (see the limitations encountered by Hainy et al. (2016) in a parameter estimation design problem for spatial extremes). For all the examples in this paper, the classification approach is significantly more time-efficient than any of the other approaches we have considered. The most crucial requirement for the applicability of the classification approach is that efficient samplers are available for all the models.

The methodology we present is rather general. We find that classification trees and random forests work very well in conjunction with the 0–1 loss. They are less suitable for loss functions that directly depend on the posterior model probability such as the multinomial deviance loss. However, one may use any other classification method that is quick and leads to accurate predictions for the application at hand. For example, logistic regression provides natural and smooth estimates for the posterior model probabilities, but it is also less flexible due to the linear form of the predictor. Generalised additive models may improve the accuracy of logistic regression at the expense of a higher computing time. Other fast classification methods include linear discriminant analysis and its extensions like mixture and flexible discriminant analysis. If a higher computing time for the classifier is acceptable and a high predictive power is desired, more elaborate methods such as neural networks may be applied. In general, for most applications it will be preferable to use a classification method where the optimal choice of the tuning parameters is insensitive to the selected design or where standard settings are available that work reasonably well in most circumstances. Otherwise, the optimal tuning parameters have to be determined for each new design, for example via cross-validation. Apart from choosing different classification methods, one may also consider different loss functions. The choice of the loss function determines the functional form of the penalty for not correctly estimating the true class. Alternatives to the 0–1 loss and multinomial deviance loss include the exponential, logit, and hinge loss functions. For an overview of all the aforementioned methods and loss functions, see Hastie et al. (2009).

One disadvantage of any simulation-based design approach is that the objective function to optimise over is stochastic. Even though the classification approach reduces the stochastic noise compared to ABC, the optimisation algorithm needs to take the noise into account. Our focus in this paper is not on optimisation, so we use a simple coordinate exchange algorithm on a discretised design space. However, our design algorithm may get stuck at suboptimal solutions if the noise is too large. We try to alleviate that problem by using parallel runs with randomly selected initial designs and by reconsidering the last few designs visited in each run, where the noise is reduced at these designs by evaluating the objective function several times. Furthermore, we employ a Gaussian process regression post-processing step where we use the data collected during the coordinate exchange procedure to train a Gaussian process in order to obtain a smooth estimate of the loss surface. This estimate of the loss surface is then minimised to find another candidate for the optimal design. Our algorithm leads to plausible optimal designs in our examples. For all our examples, the efficiencies of the optimal designs follow a reasonable trajectory as the design sizes are increased. Furthermore, the differences between the design approaches are consistent across the design sizes. For high-dimensional designs with a continuous design space and noisy objective functions, the approximate coordinate exchange algorithm (Overstall and Woods 2017) is a theoretically sound and efficient alternative. Price et al. (2018) present an ‘induced natural selection heuristic’ algorithm that can cope with moderate to high dimensions and noisy objective functions. Other possible optimisation algorithms suited for noisy objective functions in small to moderate dimensions include ‘simultaneous perturbation, stochastic approximation’ (Spall 1998) and the rather robust Nelder–Mead algorithm (Nelder and Mead 1965).

For future work, we will consider extending our approach to Bayesian parameter estimation designs. Another possibility is to attempt to utilize our classification approach within a sequential design setting in the spirit of Kleinegesse et al. (2020).

## Supplementary Information

Below is the link to the electronic supplementary material.

Supplementary file 1 (pdf 1177 KB)
