# Supplementary material for: Optimal Bayesian design for model discrimination via classification
Source: Stat Comput. 2022 Feb 22;32(2):25. doi: 10.1007/s11222-022-10078-2 (PMC8924111; doi:10.1007/s11222-022-10078-2)
Supplement: Supplementary file 1 — (pdf 1177 KB) [file 11222_2022_10078_MOESM1_ESM.pdf]

# Supplementary Materials for "Optimal Bayesian Design for Model Discrimination via Classification"

February 13, 2022

# Contents

**1 Properties and Estimation of CARTs and Random Forests 3**

**2 Modification of Coordinate Exchange Algorithm 6**

**3 Computational Performance Measures for Examples in Main Paper 8**

**4 Additional Details and Results for Epidemiological Example 14**

4.1 Prior Distributions . . . . . . . . . . . . . . . . . . . . . . . . . . . . 14

4.2 Optimal Designs for $n = 4$ and $n = 5$ . . . . . . . . . . . . . . . . . . . 14

4.3 Misclassification Matrices . . . . . . . . . . . . . . . . . . . . . . . . . 15

**5 Additional Details and Results for the Two-model Epidemiological Example 18**

5.1 Model Description and Likelihood Functions . . . . . . . . . . . . . . . 18

5.2 Approximating the Marginal Likelihood . . . . . . . . . . . . . . . . . . 19

5.3 Further Results . . . . . . . . . . . . . . . . . . . . . . . . . . . . . . 20

**6 Additional Details and Results for Macrophage Example 25**

6.1 Models and Prior Distributions . . . . . . . . . . . . . . . . . . . . . . 25

6.2 Optimal Designs . . . . . . . . . . . . . . . . . . . . . . . . . . . . . 28

6.3 Misclassification Matrix . . . . . . . . . . . . . . . . . . . . . . . . . 28

**7 Logistic Regression Example 30**

**8 Spatial Extremes Example 38**

8.1 Models . . . . . . . . . . . . . . . . . . . . . . . . . . . . . . . . . . 38

8.2 Summary Statistics . . . . . . . . . . . . . . . . . . . . . . . . . . . . 40

8.3 Bayesian Inference for Spatial Extremes Models . . . . . . . . . . . . . 41

8.4 Settings and Results . . . . . . . . . . . . . . . . . . . . . . . . . . . 42

# 1 Properties and Estimation of CARTs and Random Forests

The CART algorithm generates a binary tree where each internal node consists of one binary rule that involves exactly one of the features, e.g., $y_3 < 10$. The feature space is split recursively at the internal nodes according to the binary rules, thereby creating a partition of the feature space consisting of hyperrectangles aligned along the feature axes. Each terminal node or leaf contains all the observations in the training sample which fall into the associated hyperrectangle. The hyperrectangle region of the feature space associated to a leaf is defined by the binary rules in the nodes leading to that leaf. For a classification tree, the class label which is assigned to a particular region of the feature space is determined by majority vote of the training samples in the corresponding leaf. The class proportions of the training samples in a leaf can be used to obtain crude estimates of the posterior class probabilities for observations falling into the associated feature space region.

Trees are constructed recursively beginning at the root. Each leaf contains those training samples that meet all the conditions leading down the path from the root to that leaf. If no stopping criterion is met and the leaf's sample contains more than one distinctive feature value, the leaf is split into two daughter nodes and becomes an internal node. To that end, the binary rule that splits the sample at the node into two subsets for the two new leaves has to be determined. The feature variable and the split point are selected such that a given criterion is minimised across all subsets. For classification, the default criterion of node impurity used for growing the tree is the Gini index $\sum_{m=1}^{K} \hat{p}_m(1 - \hat{p}_m)$, where $\hat{p}_m$ is the proportion of training samples from class $m$ in the node.

As noted for example by Hastie et al. (2009), fully grown trees, where no further splits are possible, usually overfit the data. Therefore, one might stop earlier and define a minimum size of a node or a parent node. More preferably, one can grow a full tree and prune it afterwards according to a cost-complexity criterion that incorporates the node impurities and the number of terminal nodes. For an efficient algorithm to find the optimal pruned tree see Breiman et al. (1984). The optimal choice of the minimum node size or the tuning parameters for cost-complexity pruning can be determined by cross-validation.

Exploiting the similarities between trees and nearest neighbour classifiers, Breiman et al. (1984) show that the misclassification error rate of a fully grown tree is bounded above by twice the Bayes error rate, which has been shown for 1-nearest neighbour classification by Cover and Hart (1967). It also follows from Breiman et al. (1984) that the misclassification error rate of a classification tree attains the Bayes error rate as the sample size tends to infinity.

The CART algorithm automatically assumes equal prior class probabilities, even if the training sample is not balanced. This is achieved by dividing the class counts in the

leaves by the overall class counts in the training sample. Therefore, a given leaf is classified as

$$\arg\max_{m\in\{1,\dots,K\}} \frac{N_m(\text{leaf})}{N_m(\text{root})}, \tag{1.1}$$

where $N_m(\text{leaf})$ and $N_m(\text{root})$ are the number of observations from class $m$ in the leaf and in the entire training sample, respectively. One may switch off this mechanism if the training sample reflects the true prior class probabilities. It is also possible to provide user-defined prior class probabilities. In that case the fractions in (1.1) are multiplied by these user-defined prior probabilities.

Due to the recursive nature of their construction, trees exhibit a high variance. A suboptimal split at a top node affects the whole tree structure below that node, so slight changes in the data might lead to widely different trees. To reduce the variance, an ensemble method called *bagging* was proposed by Breiman (1996).

Bagging means to draw $B$ bootstrap samples from the training sample and to apply the classification method to each bootstrap sample. As a result, one obtains $B$ different classifiers trained on the $B$ bootstrap samples. The class of a new observation $\boldsymbol{y}_*$ is predicted by casting a majority vote among the class predictions returned by the $B$ classifiers. Bagging has been shown to be particularly useful for classification methods that are unstable and exhibit a high variance such as trees and neural networks, where bagging can lead to a substantial reduction of the variance.

An ensemble of bagged trees might be highly correlated, which has a negative effect on the variance of the bagged predictor. To reduce the variance further, *random forests* (Breiman, 2001) seek to de-correlate the trees by considering only a random subset of the feature variables for splitting the tree at each node when the trees are grown. For classification, the default setting is to consider $\lfloor\sqrt{p}\rfloor$ variables at each node, where $p$ is the total number of feature variables. The random selection of feature subsets reduces the correlation between the trees but it also increases the bias of the trees. On the other hand, the trees used in random forests are normally not pruned, and unpruned trees have less bias than pruned trees.

Random forests are able to account for overfitting when computing the misclassification error rate without the need to employ cross-validation or to generate a separate test set. Each tree is constructed from a bootstrap sample of the training set. The bootstrap samples are drawn from the training set with replacement. It follows that about one third of the training set is omitted in each bootstrap sample. It is therefore possible to make predictions for each training sample $\boldsymbol{y}_i$ based on those trees where $\boldsymbol{y}_i$ does not appear. These *out-of-bag* class predictions can then be used to estimate the misclassification error rate. Out-of-bag estimation is qualitatively similar to leave-one-out cross-validation.

Random forests also provide estimates for the posterior model probabilities $p(m|\boldsymbol{y}, \boldsymbol{d})$. The estimates are formed by simply averaging the posterior model probability estimates obtained from the trees in the forest. Due to the averaging, the posterior model proba-

bility estimates of the random forest are much more stable than those given by a single tree.

Unfortunately, there are some difficulties when trying to estimate the expected multinomial deviance loss by classification trees or random forests using cross-validation, independent test samples or out-of-bag class predictions. For a single tree, the lack of smoothness of its posterior model probability estimates means that in an independent test sample there are almost certainly some observations for which the estimated posterior model probability of the true model is 0. Therefore, minus the logarithm of the posterior model probability is $\infty$ and the expected multinomial deviance loss is also $\infty$. When evaluating a random forest on a test sample or when using out-of-bag class predictions, it is also very likely that some probability estimates are 0. In our examples, we therefore set the estimated posterior model probability to a value of $\varepsilon = 0.001$ whenever the posterior model probability is estimated to be 0. The lower the value of $\varepsilon$, the higher the variance of the expected loss estimate, because it becomes very sensitive to the number of posterior model probabilities estimated to be 0. In our experience, setting $\varepsilon$ to 0.001 was striking a good balance between being reasonably close to 0 while not exhibiting excessive variability.

For all our examples except the spatial extremes example, we use the Matlab functions `fitctree` and `TreeBagger` to train classification trees and random forests, respectively. We mostly use the default settings of those functions. That is, for classification trees the maximum number of splits is set to the sample size − 1, the minimum leaf size is 1 and the minimum internal node size is 10. This leads to rather deep trees. The trees are not pruned. The default settings of `TreeBagger` amount to following the standard methodology of random forests as outlined in this section. The random forests we employ are generally made up of 100 trees and utilise out-of-bag class predictions.

## 2 Modification of Coordinate Exchange Algorithm

---

**Algorithm 1:** Modification of coordinate exchange algorithm (one parallel instance)

---

**Input:** Set of available design points $\mathcal{A}$; initial design $\boldsymbol{d} = \{d_1, \ldots, d_n\}$ consisting of $n = \text{card}(\boldsymbol{d})$ design points; function `estimate_loss`$(\boldsymbol{d})$ that estimates the expected loss for a given design $\boldsymbol{d}$; numbers $p$ and $q$: for the last (at most) $p$ designs visited, the expected loss is estimated $q$ times.

**Output:** Set $\mathcal{V}_{\text{GP}}$ containing the last designs visited; set $\mathcal{L}$ containing $q$ expected loss value estimates for each design in $\mathcal{V}_{\text{GP}}$; preliminary optimal design $\boldsymbol{d}^*_{\text{CE}}$ after running one instance of the modified coordinate exchange algorithm and the corresponding expected loss value $l^*_{\text{CE}}$.

`swaps` = **true**;<br>
`loss` = `estimate_loss`$(\boldsymbol{d})$;<br>
No designs visited so far: $\mathcal{V} = \{\}$;<br>
**while** *swaps* **do**<br>
    `swaps` = **false**;<br>
    **for** $i = 1$ **to** $n$ **do**<br>
        Determine the set of candidate design points $\mathcal{C} \subseteq \mathcal{A}$;<br>
        $m = \text{card}(\mathcal{C})$;<br>
        Clear `lossvec`;<br>
        **for** $j = 1$ **to** $m$ **do**<br>
            $\boldsymbol{d}^{\text{try}} = \boldsymbol{d}$;<br>
            Replace element $i$ of $\boldsymbol{d}^{\text{try}}$ with element $j$ of $\mathcal{C}$;<br>
            `lossvec`$[j]$ = `estimate_loss`$(\boldsymbol{d}^{\text{try}})$;<br>
        **end for**<br>
        Let `minloss` = min(`lossvec`) and $k$ be the index for which `lossvec`$[k]$ is equal to `minloss`;<br>
        **if** `minloss` < `loss` **then**<br>
            Replace element $i$ of $\boldsymbol{d}$ with element $k$ of $\mathcal{C}$;<br>
            `loss` = `minloss`;<br>
            `swaps` = **true**;<br>
            Add $\boldsymbol{d}$ to $\mathcal{V}$, the history of designs visited so far;<br>
        **end if**<br>
    **end for**<br>
**end while**<br>
Let $h = \text{card}(\mathcal{V})$ be the number of designs visited, where $\mathcal{V} = \{\boldsymbol{d}_1, \ldots, \boldsymbol{d}_h\}$;<br>
Let $r = \min(h, p)$;<br>
Let $\mathcal{L} = \{\}$ and $\mathcal{AL} = \{\}$;<br>
**for** $i = 1$ **to** $r$ **do**<br>
    Clear `lossvec`;<br>
    **for** $j = 1$ **to** $q$ **do**<br>
        `lossvec`$[j]$ = `estimate_loss`$(\boldsymbol{d}_{h-i+1})$;<br>
    **end for**<br>
    Add `lossvec` as $i$th element to $\mathcal{L}$;<br>
    Add mean(`lossvec`) as $i$th element to $\mathcal{AL}$;<br>
**end for**<br>
Let $s$ be the index of the smallest element in $\mathcal{AL}$ and $l^*_{\text{CE}}$ be the corresponding value;<br>
Return $\boldsymbol{d}^*_{\text{CE}} = \boldsymbol{d}_{h-s+1}$, $l^*_{\text{CE}}$, $\mathcal{V}_{\text{GP}} = \{\boldsymbol{d}_h, \boldsymbol{d}_{h-1}, \ldots, \boldsymbol{d}_{h-r+1}\}$, $\mathcal{L}$;

---

**Algorithm 2:** Gaussian process regression post-processing step

**Input:** Sets of visited designs $\mathcal{V}_{\text{GP},i}$ and sets of corresponding expected loss estimates $\mathcal{L}_i$ ($q$ values for each design in $\mathcal{V}_{\text{GP},i}$) for $i = 1, \ldots, I$ parallel runs of the modified coordinate exchange algorithm (Algorithm 1); preliminary optimal designs $\boldsymbol{d}^*_{\text{CE},i}$ and corresponding estimated expected loss values $l^*_{\text{CE},i}$ for $i = 1, \ldots, I$ parallel runs of Algorithm 1; function `estimate_loss`($\boldsymbol{d}$) that estimates the expected loss for a given design $\boldsymbol{d}$.

**Output:** Overall optimal design $\boldsymbol{d}^*$.

Combine sets of visited designs $\mathcal{V}_{\text{GP},i}$ for all $i = 1, \ldots, I$ parallel runs into one set $\mathcal{V}_{\text{GP}}$. Do the same for the sets of expected loss estimates $\mathcal{L}_i$ and combine them into $\mathcal{L}$;
Train Gaussian process with the expected loss values in $\mathcal{L}$ as (univariate) response variable and the visited designs $\mathcal{V}_{\text{GP}}$ as predictors (each design is repeated $q$ times);
Find the minimum value of the predictive mean function of the Gaussian process over the design space using some generic optimisation function. Let the design at the minimum be denoted by $\boldsymbol{d}^*_{\text{GP}}$;
Set $\boldsymbol{d}^*_{\text{CE}}$ to the design $\boldsymbol{d}^*_{\text{CE,i}}$ from parallel run $i$ with the lowest value for $l^*_{\text{CE},i}$;
Clear `lossvec_CE`, `lossvec_GP`;
**for** $j = 1$ **to** 100 **do**
    `lossvec_CE`[$j$] = `estimate_loss`($\boldsymbol{d}^*_{\text{CE}}$);
    `lossvec_GP`[$j$] = `estimate_loss`($\boldsymbol{d}^*_{\text{GP}}$);
**end for**
**if** mean(`lossvec_GP`) < mean(`lossvec_CE`) **then**
    Return $\boldsymbol{d}^* = \boldsymbol{d}^*_{\text{GP}}$;
**else**
    Return $\boldsymbol{d}^* = \boldsymbol{d}^*_{\text{CE}}$;
**end if**

In all our examples we set $p = 6$ and $q = 10$.

Algorithm 1 can be run in parallel for different initial designs $\boldsymbol{d}$ to account for multi-modality and local optima. We conduct 20 parallel runs in all our examples.

The selection of the candidate design points in Line 7 of Algorithm 1 depends on the example. For the logistic regression and the macrophage example, there is no restriction and $\mathcal{C} = \mathcal{A}$. For the other examples, the current design points $d_1, \ldots, d_n$ in $\boldsymbol{d}$ have to be excluded since each design point can only be selected once. Furthermore, for the spatial extremes example we only consider design points with the same x- or y-coordinate as the current design point.

The sets of best designs found in each of the parallel runs of Algorithm 1 and their associated estimated expected loss values are combined and used as inputs for Algorithm 2. In Algorithm 2, a Gaussian process (GP; see, e.g., Rasmussen and Williams, 2006) is trained on the combined data from all the parallel runs in order to obtain a smooth estimate of the expected loss surface by means of the predictive mean function of the GP. The predictive mean function is minimised and a new candidate for the optimal design is obtained. Since the predictive variance is relatively high in our examples, we compare this design to the best design found through Algorithm 1 without the GP post-processing step of Algorithm 2. To reduce the uncertainty for this comparison, we estimate the expected loss 100 times at each of the two designs and take the design

with the lower average expected loss value as the overall optimal design. We do not perform the GP post-processing step for the spatial extremes example.

For Gaussian process regression, we use the default settings of the Matlab function `fitrgp` except that all the predictors are standardised. The default kernel function used is the squared exponential kernel and a constant GP prior mean is assumed. To find the optimal value for the initial value of the prior noise variance parameter, Bayesian optimisation is conducted with respect to the cross-validation loss.

For finding the minimum of the GP's predictive mean function, we use the Nelder-Mead simplex algorithm (Nelder and Mead, 1965). Restrictions of the design space are considered by employing suitable transformations. For example, design points with the restriction $d_i \in (a, b]$ are transformed by the logit transformation to $\tilde{d}_i = \log\{z_i/(1-z_i)\}$, where $z_i = (d_i - a)/(b - a)$.

# 3 Computational Performance Measures for Examples in Main Paper

In this section, we provide some measures of computational performance for the design search algorithms used for the three examples in Section 4 of the main paper. As explained in Section 2, we ran Algorithm 1 twenty times in parallel, so there is a distribution of runtimes to consider. We focus on the exchange part of Algorithm 1 (lines 4 to 23), because this is usually the most time-consuming part. However, it is not sensible to just compare the distributions of runtimes of the exchange part because the number of sweeps through the design grid until the algorithm converges (i.e., the number of passes through the `while`-loop) is random. Therefore, in Tables 1 to 4 we provide the distributions for the runtimes per sweep for all the examples in Section 4.1 of the main paper. More precisely, we state the mean and the standard deviation of the runtime per sweep over the parallel runs. As expected, one can see that these distributions exhibit little variation. The reason is that the number of calls of the `estimate_loss` function in a sweep through the design grid is fixed for any given example and design configuration. Within any call to `estimate_loss`, the simulated sample sizes are fixed (for details see the example settings for the respective models in Section 4 of the main paper). The sample sizes for trees and random forests are always the same, so differences in runtimes can solely be attributed to the classification method. In general, for all our examples simulation is rather efficient, so the simulation effort is only a minor fraction of the total runtime. This is also true for ABC, where sorting the reference table for each draw from the outer sample is much more time-consuming than creating the reference table itself (see also Section 4.1.4 in the main paper).

It is interesting to note that there do not seem to be any systematic differences between the distributions of the number of sweeps between the different methods. Therefore, it is entirely sufficient to consider the runtimes per sweep or runtimes per call when

analysing the differences between the methods.

In our examples, it was about four to five times faster to use cross-validated trees than to use random forests when the data dimension is small. However, as the data dimension increases, the tree method loses some of that advantage (see Tables 3 and 4). Note that in the macrophage example the data consist of the various observed cell proportions at each design point and are therefore quite high-dimensional despite the low dimensionality of the designs. Furthermore, the higher the dimension, the bigger the advantage of the designs found through random forest classification in terms of discriminatory performance (see, e.g., Figure 5 in this document). Therefore, for higher dimensions the recommendation is to use random forests.

Both classification approaches are many times faster than the other approaches investigated in Table 1 (ABC) and Table 2 (likelihood-based). Note that this is despite the relatively small simulation sizes for the outer Monte Carlo samples from the prior predictive distribution that we used for ABC (sample size 2000) as well as for the likelihood-based approach (sample size 800) to keep the runtimes within a tolerable range. These small outer sample sizes led to a considerably larger noise in the expected loss estimates for those two approaches compared to the classification approaches (see Figure 1 in the main paper and Figure 3 in this document).

Runtimes are machine- and implementation-specific and should therefore be taken with caution. However, Tables 1 to 4 can still give some clues on the relative efficiency of the different methods. All our examples were run on an SGI UV 3000 global shared memory system from Hewlett Packard Enterprises. It uses 12-core processors of type Intel Xeon E5-4650V3 that operate on 2.8 GHz and have an L3 cache of 30 MB.

We do not further analyse Algorithm 2.

**Table 1:** Several performance indicators for the infectious disease example of Section 4.1 from the main paper: number of sweeps ($s$) of coordinate exchange algorithm through design grid (minimum, median, maximum over 20 parallel runs), calls ($c$) to loss estimation procedure per sweep ($s$), mean and standard deviation of runtime ($r$) per sweep ($s$) over all parallel runs (in minutes).

| $n$ | Method | min($s$) | med($s$) | max($s$) | $c/s$ | mean($r/s$) | std($r/s$) |
|---|---|---|---|---|---|---|---|
| 1 | Tree CV 01L | 1 | 2 | 4 | 39 | 0.8 | 0.03 |
| | Tree CV MDL | 2 | 2 | 4 | 39 | 0.3 | 0.01 |
| | RF 01L | 2 | 2 | 4 | 39 | 3.3 | 0.18 |
| | RF MDL | 2 | 2 | 3 | 39 | 3.1 | 0.16 |
| | ABC 01L | 1 | 2 | 3 | 39 | 33.2 | 1.75 |
| | ABC MDL | 2 | 2 | 4 | 39 | 33.6 | 1.92 |
| 2 | Tree CV 01L | 2 | 2 | 5 | 76 | 1.6 | 0.05 |
| | Tree CV MDL | 2 | 3 | 4 | 76 | 0.8 | 0.04 |
| | RF 01L | 2 | 3 | 6 | 76 | 8.1 | 0.30 |
| | RF MDL | 2 | 3 | 6 | 76 | 7.4 | 0.30 |
| | ABC 01L | 2 | 3 | 5 | 76 | 95.2 | 4.88 |
| | ABC MDL | 2 | 2.5 | 5 | 76 | 92.7 | 6.57 |
| 3 | Tree CV 01L | 2 | 2.5 | 6 | 111 | 2.7 | 0.10 |
| | Tree CV MDL | 2 | 2 | 5 | 111 | 1.5 | 0.05 |
| | RF 01L | 2 | 2.5 | 6 | 111 | 12.1 | 0.51 |
| | RF MDL | 2 | 3 | 5 | 111 | 12.1 | 0.27 |
| | ABC 01L | 2 | 3 | 5 | 111 | 179.0 | 5.81 |
| | ABC MDL | 2 | 3 | 5 | 111 | 176.9 | 5.50 |
| 4 | Tree CV 01L | 2 | 3 | 5 | 144 | 4.0 | 0.18 |
| | Tree CV MDL | 2 | 3 | 5 | 144 | 2.3 | 0.08 |
| | RF 01L | 2 | 2.5 | 4 | 144 | 17.1 | 1.18 |
| | RF MDL | 2 | 3 | 4 | 144 | 16.4 | 1.39 |
| | ABC 01L | 2 | 2 | 4 | 144 | 283.9 | 7.94 |
| | ABC MDL | 2 | 3 | 5 | 144 | 281.4 | 8.88 |
| 5 | Tree CV 01L | 2 | 2 | 5 | 175 | 5.5 | 0.32 |
| | Tree CV MDL | 2 | 3 | 7 | 175 | 3.0 | 0.16 |
| | RF 01L | 2 | 3 | 5 | 175 | 25.9 | 2.39 |
| | RF MDL | 2 | 3 | 5 | 175 | 23.8 | 1.09 |
| | ABC 01L | 2 | 3 | 5 | 175 | 414.6 | 13.36 |
| | ABC MDL | 2 | 3 | 5 | 175 | 408.4 | 16.98 |

**Table 2:** Several performance indicators for the two-model infectious disease example of Section 4.2 from the main paper (lower-dimensional designs): number of sweeps ($s$) of coordinate exchange algorithm through design grid (minimum, median, maximum over 20 parallel runs), calls ($c$) to loss estimation procedure per sweep ($s$), mean and standard deviation of runtime ($r$) per sweep ($s$) over all parallel runs (in minutes).

| $n_d$ | $q$ | Method | min($s$) | med($s$) | max($s$) | $c/s$ | mean($r/s$) | std($r/s$) |
|---|---|---|---|---|---|---|---|---|
| 1 | 1 | Tree CV | 2 | 2 | 2 | 19 | 0.2 | 0.01 |
| | | RF | 1 | 2 | 2 | 19 | 0.9 | 0.05 |
| | | ML | 1 | 2 | 3 | 19 | 4.9 | 0.07 |
| | 2 | Tree CV | 1 | 3 | 4 | 38 | 0.5 | 0.02 |
| | | RF | 2 | 3 | 5 | 38 | 2.2 | 0.12 |
| | | ML | 1 | 3 | 4 | 38 | 17.0 | 0.26 |
| | 3 | Tree CV | 2 | 3 | 5 | 57 | 0.9 | 0.03 |
| | | RF | 2 | 3 | 5 | 57 | 3.8 | 0.16 |
| | | ML | 2 | 3 | 5 | 57 | 36.0 | 0.46 |
| | 4 | Tree CV | 2 | 3 | 5 | 76 | 1.3 | 0.05 |
| | | RF | 2 | 2 | 4 | 76 | 5.6 | 0.22 |
| | | ML | 2 | 2.5 | 8 | 76 | 61.4 | 1.08 |
| 2 | 1 | Tree CV | 1 | 2 | 4 | 36 | 0.4 | 0.02 |
| | | RF | 2 | 2.5 | 5 | 36 | 2.0 | 0.12 |
| | | ML | 1 | 2 | 5 | 36 | 15.3 | 0.26 |
| | 2 | Tree CV | 2 | 3 | 5 | 72 | 1.2 | 0.04 |
| | | RF | 2 | 3 | 5 | 72 | 5.2 | 0.14 |
| | | ML | 2 | 3 | 5 | 72 | 56.3 | 0.43 |
| | 3 | Tree CV | 2 | 3.5 | 6 | 108 | 2.3 | 0.06 |
| | | RF | 2 | 3 | 6 | 108 | 10.1 | 0.47 |
| | | ML | 2 | 3 | 5 | 108 | 119.0 | 1.59 |
| | 4 | Tree CV | 2 | 3 | 7 | 144 | 3.5 | 0.07 |
| | | RF | 2 | 3 | 6 | 144 | 13.6 | 0.54 |
| | | ML | 2 | 3 | 5 | 144 | 214.8 | 1.89 |
| 3 | 1 | Tree CV | 2 | 3 | 4 | 51 | 0.7 | 0.03 |
| | | RF | 2 | 2.5 | 6 | 51 | 3.1 | 0.15 |
| | | ML | 2 | 2.5 | 4 | 51 | 29.2 | 0.33 |
| | 2 | Tree CV | 2 | 3 | 4 | 102 | 2.0 | 0.07 |
| | | RF | 2 | 3 | 9 | 102 | 9.5 | 0.34 |
| | | ML | 2 | 3 | 5 | 102 | 111.7 | 1.07 |
| 4 | 1 | Tree CV | 2 | 3 | 5 | 64 | 1.0 | 0.04 |
| | | RF | 2 | 2 | 5 | 64 | 4.2 | 0.26 |
| | | ML | 2 | 3 | 6 | 64 | 46.6 | 0.43 |
| | 2 | Tree CV | 2 | 3 | 6 | 128 | 2.8 | 0.10 |
| | | RF | 2 | 4 | 6 | 128 | 11.8 | 0.35 |
| | | ML | 2 | 3 | 5 | 128 | 180.9 | 1.26 |

**Table 3:** Several performance indicators for the two-model infectious disease example of Section 4.2 from the main paper (higher-dimensional designs): number of sweeps ($s$) of coordinate exchange algorithm through design grid (minimum, median, maximum over 20 parallel runs), calls ($c$) to loss estimation procedure per sweep ($s$), mean and standard deviation of runtime ($r$) per sweep ($s$) over all parallel runs (in minutes).

| $n_d$ | $q$ | Method | min($s$) | med($s$) | max($s$) | $c/s$ | mean($r/s$) | std($r/s$) |
|---|---|---|---|---|---|---|---|---|
| 1 | 12 | Tree CV | 2 | 3 | 6 | 228 | 7.5 | 0.19 |
| | | RF | 2 | 3 | 8 | 228 | 25.5 | 0.71 |
| | 24 | Tree CV | 2 | 3 | 8 | 456 | 25.3 | 0.50 |
| | | RF | 2 | 3.5 | 6 | 456 | 63.1 | 1.47 |
| | 36 | Tree CV | 2 | 2.5 | 9 | 684 | 61.4 | 1.26 |
| | | RF | 2 | 4 | 8 | 684 | 110.8 | 4.10 |
| | 48 | Tree CV | 2 | 3 | 7 | 912 | 114.7 | 2.52 |
| | | RF | 2 | 3 | 6 | 912 | 169.8 | 5.30 |
| 2 | 6 | Tree CV | 2 | 3 | 7 | 216 | 7.1 | 0.27 |
| | | RF | 2 | 4 | 7 | 216 | 23.7 | 0.66 |
| | 12 | Tree CV | 2 | 2.5 | 5 | 432 | 24.0 | 0.81 |
| | | RF | 2 | 3 | 6 | 432 | 58.1 | 1.91 |
| | 18 | Tree CV | 2 | 3 | 7 | 648 | 51.6 | 1.04 |
| | | RF | 2 | 3 | 6 | 648 | 100.8 | 3.57 |
| | 24 | Tree CV | 2 | 2.5 | 5 | 864 | 94.7 | 2.94 |
| | | RF | 2 | 3 | 7 | 864 | 153.4 | 6.85 |
| 3 | 4 | Tree CV | 2 | 3 | 5 | 204 | 6.5 | 0.30 |
| | | RF | 2 | 3 | 7 | 204 | 22.2 | 0.62 |
| | 8 | Tree CV | 2 | 3 | 6 | 408 | 21.3 | 0.41 |
| | | RF | 2 | 4 | 7 | 408 | 53.8 | 1.15 |
| | 12 | Tree CV | 2 | 3 | 7 | 612 | 44.0 | 1.27 |
| | | RF | 2 | 4 | 7 | 612 | 93.4 | 3.26 |
| | 16 | Tree CV | 2 | 3 | 4 | 816 | 78.4 | 1.68 |
| | | RF | 2 | 3.5 | 5 | 816 | 138.9 | 5.88 |
| 4 | 3 | Tree CV | 2 | 3 | 6 | 192 | 5.9 | 0.10 |
| | | RF | 2 | 3 | 8 | 192 | 20.7 | 0.60 |
| | 6 | Tree CV | 2 | 3 | 6 | 384 | 17.9 | 0.58 |
| | | RF | 2 | 4 | 8 | 384 | 49.0 | 1.59 |
| | 9 | Tree CV | 2 | 3 | 5 | 576 | 35.0 | 1.14 |
| | | RF | 2 | 4 | 7 | 576 | 85.4 | 2.78 |
| | 12 | Tree CV | 2 | 2.5 | 6 | 768 | 61.8 | 1.36 |
| | | RF | 2 | 3.5 | 6 | 768 | 127.4 | 5.16 |

**Table 4:** Several performance indicators for the macrophage example of Section 4.3 from the main paper: number of sweeps ($s$) of coordinate exchange algorithm through design grid (minimum, median, maximum over 20 parallel runs), calls ($c$) to loss estimation procedure per sweep ($s$), mean and standard deviation of runtime ($r$) per sweep ($s$) over all parallel runs (in minutes).

| $n$ | Method | min($s$) | med($s$) | max($s$) | $c/s$ | mean($r/s$) | std($r/s$) |
|---|---|---|---|---|---|---|---|
| 1 | Tree CV | 2 | 3 | 6 | 55 | 10.4 | 0.6 |
| | RF | 2 | 4 | 5 | 55 | 18.6 | 9.5 |
| 2 | Tree CV | 2 | 3 | 4 | 95 | 40.5 | 8.1 |
| | RF | 2 | 3 | 6 | 95 | 53.4 | 19.4 |
| 3 | Tree CV | 2 | 3 | 5 | 135 | 72.6 | 18.5 |
| | RF | 2 | 3 | 6 | 135 | 82.0 | 14.0 |
| 4 | Tree CV | 2 | 3 | 6 | 175 | 108.9 | 24.4 |
| | RF | 2 | 3 | 8 | 175 | 159.0 | 33.2 |
| 5 | Tree CV | 2 | 3 | 5 | 215 | 156.1 | 57.1 |
| | RF | 2 | 3 | 4 | 215 | 176.3 | 37.0 |

# 4 Additional Details and Results for Epidemiological Example

## 4.1 Prior Distributions

The prior distributions for the four epidemiological Markov process models of Section 4.1 are given in Table 5.

**Table 5:** The prior distributions considered for the infectious disease example of Section 4.1. Here $\mathcal{LN}(\mu, \sigma)$ denotes the lognormal distribution with location $\mu$ and scale $\sigma$. $\mathcal{E}(\eta)$ denotes the exponential distribution with rate $\eta$.

| Model Number | Parameter | Prior |
|---|---|---|
| Model 1 | $b_1^{(1)}$ | $\mathcal{LN}(-0.48, 0.09)$ |
| Model 2 | $b_1^{(2)}$ | $\mathcal{LN}(-1.1, 0.16)$ |
| | $b_2^{(2)}$ | $\mathcal{LN}(-4.5, 0.4)$ |
| Model 3 | $b_1^{(3)}$ | $\mathcal{LN}(-0.54, 0.15)$ |
| | $\gamma^{(3)}$ | $\mathcal{E}(0.01)$ |
| Model 4 | $b_1^{(4)}$ | $\mathcal{LN}(-1.34, 0.41)$ |
| | $b_2^{(4)}$ | $\mathcal{LN}(-4.26, 0.25)$ |
| | $\gamma^{(4)}$ | $\mathcal{E}(0.01)$ |

## 4.2 Optimal Designs for $n = 4$ and $n = 5$

**Table 6:** Optimal designs obtained by tree classification (cross-validated), random forest classification (using out-of-bag class predictions), and ABC approaches under the 0–1 loss (01L) or multinomial deviance loss (MDL) ($n = 4$ and 5) for the infectious disease example. The equidistant designs are also shown.

| Method/Loss | $n = 4$ | | | | $n = 5$ | | | | |
|---|---|---|---|---|---|---|---|---|---|
| Tree 01L | 0.750 | 4.250 | 9.750 | 10.000 | 0.910 | 4.304 | 8.671 | 10.000 | 10.000 |
| RF 01L | 0.750 | 4.250 | 8.500 | 9.750 | 0.750 | 4.250 | 8.250 | 9.000 | 9.250 |
| ABC 01L | 0.047 | 0.599 | 2.265 | 4.943 | 0.250 | 1.000 | 3.000 | 5.500 | 7.500 |
| Tree MDL | 0.750 | 5.000 | 9.791 | 10.000 | 0.750 | 4.750 | 9.566 | 9.750 | 10.000 |
| RF MDL | 0.720 | 4.000 | 6.268 | 10.000 | 0.750 | 4.250 | 9.566 | 9.750 | 10.000 |
| ABC MDL | 0.222 | 0.703 | 3.120 | 5.753 | 0.500 | 1.500 | 3.000 | 4.750 | 7.250 |
| Equidistant | 2.000 | 4.000 | 6.000 | 8.000 | 1.667 | 3.333 | 5.000 | 6.667 | 8.333 |

## 4.3 Misclassification Matrices

The random forest classifiers and the corresponding random samples which we use to compute the misclassification error rates in Table 3 of the main paper can also be used to compute misclassification matrices for the various optimal designs. A *misclassification* or *confusion matrix* contains for each combination of true model $m_i$ (in the rows) and predicted model $m_j$ (in the columns) the proportions of samples from true model $m_i$ that were classified as model $m_j$. In the case of random forests, the misclassification matrix is computed using out-of-bag class predictions. It provides a comprehensive picture of the classification accuracy at a given design.

For the optimal design obtained by the tree classification approach with cross-validation under the 0–1 loss, the misclassification matrices for 1 – 4 time points are shown in Figure 1. The figure suggests that it is difficult to discriminate between models 1 and 3 and also models 2 and 4. This is not surprising given that we do not observe the exposed population. Especially model 3 is most often misclassified as model 1. The misclassification matrices for the other machine learning classification approaches and loss functions are qualitatively all very similar to Figure 1.

In Figure 2, the misclassification matrices for the ABC approach under the 0–1 loss are depicted. The ABC approach leads to designs with generally lower values for the design points than the machine learning approaches (see Table 2 in the main paper and Table 6 in this document). The overall misclassification error rates are similar, but one can see that the pattern is a bit different from Figure 1. At 4 design points, model 3 is more likely to be correctly classified, but the misclassification error of models 1 and 2 increases.

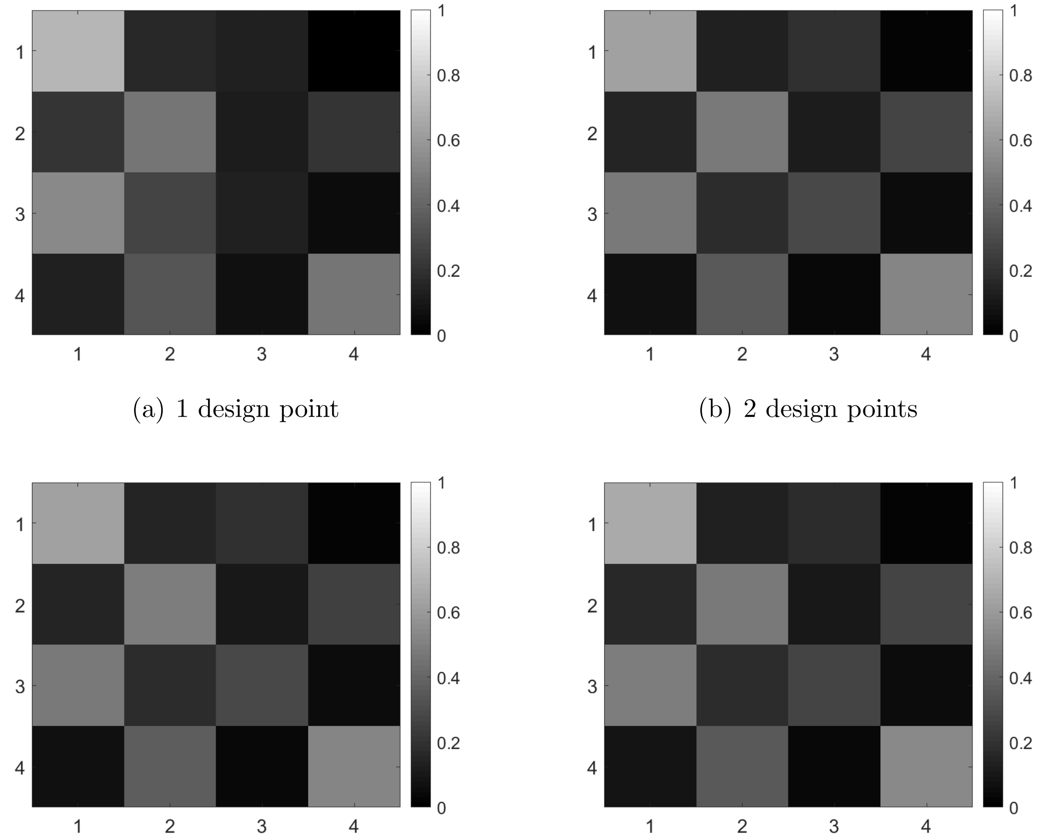

(a) 1 design point

(b) 2 design points

(c) 3 design points

(d) 4 design points

**Figure 1:** Misclassification matrices obtained for the *tree classification designs (using cross-validation)* under the *0–1 loss* for the infectious disease example. Designs for 1 – 4 observations are considered.

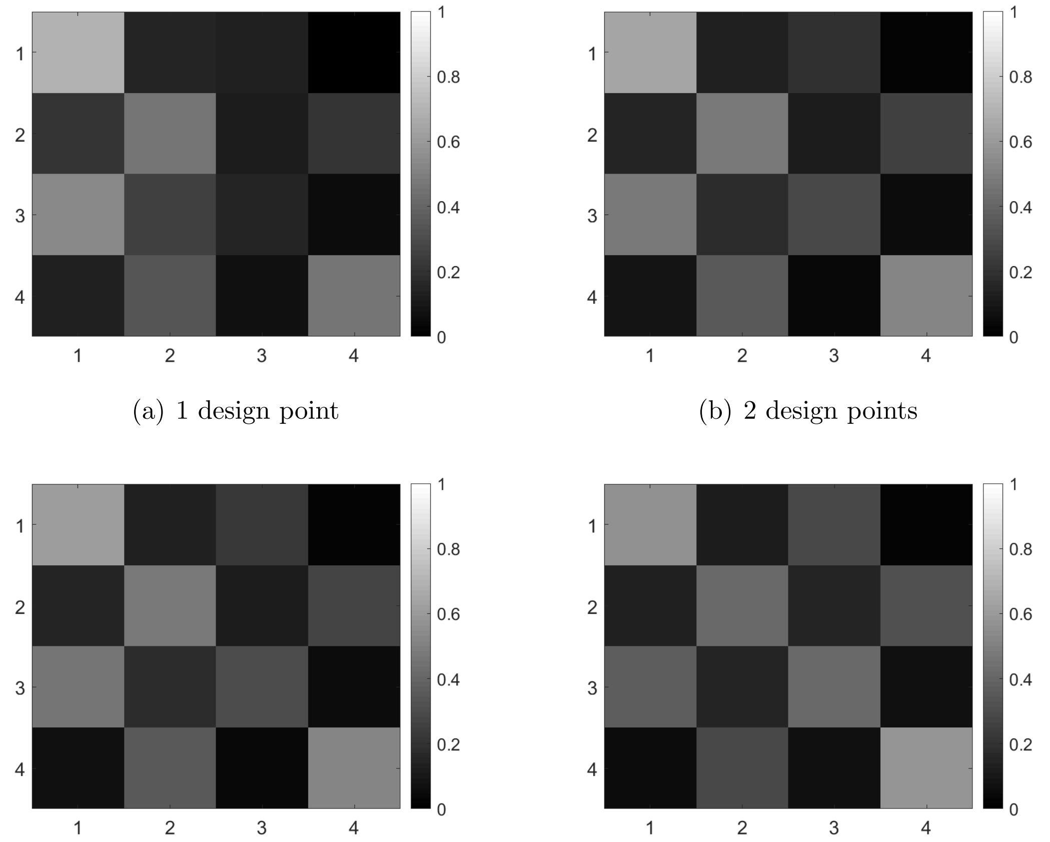


(a) 1 design point

(b) 2 design points

(c) 3 design points

(d) 4 design points

**Figure 2:** Misclassification matrices obtained for the *ABC designs* under the *0–1 loss* for the infectious disease example. Designs for 1 – 4 observations are considered.

# 5 Additional Details and Results for the Two-model Epidemiological Example

## 5.1 Model Description and Likelihood Functions

Let the design for realisation $i$ ($i = 1, \ldots, q$) be given by $\boldsymbol{d}_i = (d_{i,1}, \ldots, d_{i,n_d})$ and the overall design be given by $\boldsymbol{d} = (\boldsymbol{d}_1, \ldots, \boldsymbol{d}_q)$, where $d_{i,j}$ is the $j^{\text{th}}$ observation time for realisation $i$. Denote the observed number of infected and susceptible subjects for realisation $i$ at time $d_{i,j}$ by $I(d_{i,j}) = I_{i,j}$ and $S(d_{i,j}) = S_{i,j}$, respectively, where $S_{i,j} = N - I_{i,j}$. Collect all the $S_{i,j}$ in the vector $\boldsymbol{S}$ in the same way as the design times $d_{i,j}$ have been collected in the vector $\boldsymbol{d}$. Each $S_{i,j}$ is a discrete random variable that can assume the $N+1$ values 0 to $N$. The parameters are denoted by $\boldsymbol{\theta} = (\log(b_1), \log(b_2))$.

Since the death and SI models are continuous-time Markov processes, their likelihood functions have the form

$$p(\boldsymbol{S}|\boldsymbol{\theta}, \boldsymbol{d}) = \prod_{i=1}^{q} \prod_{j=1}^{n_d} \Pr\left(S_{i,j} |\, S_{i,j-1}, \boldsymbol{\theta}, d_{i,j-1}, d_{i,j}\right), \tag{5.1}$$

where $S_{i,0} = N$ is the number of susceptible individuals at time $d_{i,0} = 0 \;\forall\, i$ (see, e.g., Cook et al., 2008).

Let the $(N+1)$-dimensional vector $\boldsymbol{v}_{i,j|S_{i,j-1}=k}$ contain the probabilities of all the possible states of the random variable $S_{i,j}$ when the value of $S_{i,j-1}$ is known to be $k$. The $m^{\text{th}}$ element of $\boldsymbol{v}_{i,j|S_{i,j-1}=k}$ gives the probability that $S_{i,j} = m-1$ when $S_{i,j-1} = k$. Since the value of $S_{i,j-1}$ is known and therefore certain, the state probability vector at observation time $d_{i,j-1}$ reduces to $\boldsymbol{e}_{k+1}$, where $\boldsymbol{e}_m$ denotes a vector for which the $m^{\text{th}}$ element is 1 and the remaining elements are 0.

Given the vectors and notation introduced above, the transition probabilities can be written as

$$\Pr\left(S_{i,j} |\, S_{i,j-1}, \boldsymbol{\theta}, d_{i,j-1}, d_{i,j}\right) = \boldsymbol{v}^T_{i,j|S_{i,j-1}} \cdot \boldsymbol{e}_{S_{i,j}+1} = \boldsymbol{e}^T_{S_{i,j-1}+1} \boldsymbol{A}_{\boldsymbol{\theta},i,j}\, \boldsymbol{e}_{S_{i,j}+1},$$

where the matrix $\boldsymbol{A}_{\boldsymbol{\theta},i,j}$ has dimension $(N+1) \times (N+1)$ and contains the transition probabilities for all pairs of states between observation times $d_{i,j-1}$ and $d_{i,j}$. This matrix follows from the solution of the Kolmogorov forward equations and can be calculated using the matrix exponential (see Higham, 2008),

$$\boldsymbol{A}_{\boldsymbol{\theta},i,j} = \exp[(d_{i,j} - d_{i,j-1})\, \boldsymbol{G}_{\boldsymbol{\theta}}], \tag{5.2}$$

where $\boldsymbol{G}_{\boldsymbol{\theta}}$ is the infinitesimal generator matrix that is constructed from the transition rates given in Table 1 of the main paper, see, e.g., Grimmett and Stirzaker (2001), pp. 258.

Let the $N+1$ rows of the generator matrix be numbered from 0 to $N$. For the SI model, row $i$ ($i = 0, \ldots, N$) of the generator matrix is given by

$$[\boldsymbol{G_\theta}]_{i\cdot} = \Big(\underbrace{0}_{\times \max\{0,i-1\}}, \underbrace{[b_1 + b_2(N-i)]\, i}_{\times \min\{1,i\}}, \underbrace{-[b_1 + b_2(N-i)]\, i}_{\times 1}, \underbrace{0}_{\times (N-i)}\Big).$$

Setting $b_2 = 0$ for the death model, the transition probabilities can be simplified to a binomial probability mass function (see Cook et al., 2008):

$$\Pr\left(S_{i,j} \,|\, S_{i,j-1}, b_1, d_{i,j-1}, d_{i,j}\right) = \mathcal{B}\left\{S_{i,j} \,|\, S_{i,j-1},\, \exp[-b_1(d_{i,j} - d_{i,j-1})]\right\}.$$

Therefore, there is no need to numerically compute the matrix exponential for the death model, and so the likelihood function can be evaluated very quickly. However, for the SI model each of the $q \cdot n_d$ matrices $\boldsymbol{A}_{\boldsymbol{\theta},i,j}$ in the likelihood function (5.1) is obtained by numerical computation of the matrix exponential (5.2).

## 5.2 Approximating the Marginal Likelihood

To obtain

$$p(m|\boldsymbol{S}, \boldsymbol{d}) \propto p(\boldsymbol{S}|m, \boldsymbol{d})\, p(m),$$

we need to compute the marginal likelihood

$$p(\boldsymbol{S}|m, \boldsymbol{d}) = \int_{\boldsymbol{\theta}_m} p(\boldsymbol{S}|\boldsymbol{\theta}_m, m, \boldsymbol{d})\, p(\boldsymbol{\theta}_m|m)\, \mathrm{d}\boldsymbol{\theta}_m. \tag{5.3}$$

We pursue two different approaches to approximating this integral. During the optimisation procedure, we use a comparatively quick Laplace-type approximation to the marginal likelihood, see Gelman et al. (2013), p. 318. Let

$$\tilde{\boldsymbol{\theta}}_m = \arg\max_{\boldsymbol{\theta}_m} p(\boldsymbol{S}|\boldsymbol{\theta}_m, m, \boldsymbol{d})\, p(\boldsymbol{\theta}_m|m) \tag{5.4}$$

be the posterior mode of model $m$. Performing a second-order Taylor expansion of $p(\boldsymbol{S}|\boldsymbol{\theta}_m, m, \boldsymbol{d})\, p(\boldsymbol{\theta}_m|m)$ around $\tilde{\boldsymbol{\theta}}_m$ and integrating out $\boldsymbol{\theta}_m$ yields

$$p(\boldsymbol{S}|m, \boldsymbol{d}) \approx (2\pi)^{p_m/2}\, |\widetilde{\boldsymbol{\Sigma}}_{\boldsymbol{S},\tilde{\boldsymbol{\theta}}_m,\boldsymbol{d}}|^{1/2}\, p(\boldsymbol{S}|\tilde{\boldsymbol{\theta}}_m, m, \boldsymbol{d})\, p(\tilde{\boldsymbol{\theta}}_m|m), \tag{5.5}$$

where $p_m$ is the number of parameters of model $m$ and

$$\widetilde{\boldsymbol{\Sigma}}^{-1}_{\boldsymbol{S},\tilde{\boldsymbol{\theta}}_m,\boldsymbol{d}} = -\nabla_{\boldsymbol{\theta}_m} \nabla^T_{\boldsymbol{\theta}_m} \left[\log p(\boldsymbol{S}|\boldsymbol{\theta}_m, m, \boldsymbol{d}) + \log p(\boldsymbol{\theta}_m|m)\right]\Big|_{\tilde{\boldsymbol{\theta}}_m} \tag{5.6}$$

is the Hessian of the negative log-posterior evaluated at the posterior mode.

When validating the optimal designs found by the different methods, we employ generalised Gauss-Hermite quadrature (Kautsky and Elhay, 1982; Elhay and Kautsky, 1987)

with $Q$ sample points to compute the integral (5.3). As weighting kernel we use a multivariate normal density with mean and variance-covariance matrix given by the mean and twice the variance-covariance matrix, respectively, of the normal Laplace approximation to the posterior,

$$\omega(\boldsymbol{\theta}_m) = \mathcal{N}(\boldsymbol{\theta}_m | \tilde{\boldsymbol{\theta}}_m, 2\, \widetilde{\boldsymbol{\Sigma}}_{\boldsymbol{S},\tilde{\boldsymbol{\theta}}_m,\boldsymbol{d}}),$$

where $\tilde{\boldsymbol{\theta}}_m$ is given by (5.4) and $\widetilde{\boldsymbol{\Sigma}}_{\boldsymbol{S},\tilde{\boldsymbol{\theta}}_m,\boldsymbol{d}}$ is given by (5.6). Using this weighting kernel, we expect that many sample points are in relevant regions where the integrand has high mass. In the bivariate case, determining the sample points involves two steps, see Jäckel (2005). First, all combinations of sample points resulting from applying the standard univariate Gauss-Hermite quadrature rule to each dimension are considered. The sample weights are simply computed by multiplying the univariate weights. To account for the correlation and different scaling and location implied by the multivariate normal weighting kernel, the sample points are then transformed accordingly based on a spectral decomposition of the variance-covariance matrix, seeking to align the diagonals of the rectangle of sample points to the principal axes of the confidence ellipsoid. Furthermore, for the two-parameter SI model we drop sample points below a weight of $w_1 \cdot w_{\lfloor(\sqrt{Q}+1)/2\rfloor}/\sqrt{Q}$, where $\sqrt{Q}$ is the number of univariate sample points of the Gauss-Hermite quadrature rule and $w_i$ denotes the weight for the $i$th ordered univariate sample point.

After obtaining the $Q$ sample points $\boldsymbol{\theta}_{m,i}$ and quadrature weights $w_i$ ($i = 1, \ldots, Q$) according to the quadrature rule, the marginal likelihood can be approximated by

$$p(\boldsymbol{S}|m, \boldsymbol{d}) \approx \sum_{i=1}^{Q} w_i \, \frac{p(\boldsymbol{S}|\boldsymbol{\theta}_{m,i}, m, \boldsymbol{d})\, p(\boldsymbol{\theta}_{m,i}|m)}{\mathcal{N}(\boldsymbol{\theta}_{m,i}|\tilde{\boldsymbol{\theta}}_m, 2\, \widetilde{\boldsymbol{\Sigma}}_{\boldsymbol{S},\tilde{\boldsymbol{\theta}}_m,\boldsymbol{d}})}. \tag{5.7}$$

## 5.3 Further Results

Figure 3 shows the estimated expected 0–1 loss surface for the one-dimensional design obtained by the different approaches using the simulation sizes we used for the design search. The comparatively high volatility of the expected 0–1 loss under the likelihood-based approach is evident from Figure 3. To create Figure 3 on our computer, it took about 17 seconds for the tree classification approach, about 2.7 minutes for the random forest classification approach, but more than 18 minutes for the likelihood-based approach despite the low data dimension and the much smaller prior predictive sample size.

Figures 4 (lower-dimensional designs) and 5 (higher-dimensional designs) display the distributions of posterior model probabilities for samples of size 2K (1K per model) from the prior predictive distribution at the various optimal designs found for all the dimension settings and the different methods. We also include equispaced designs

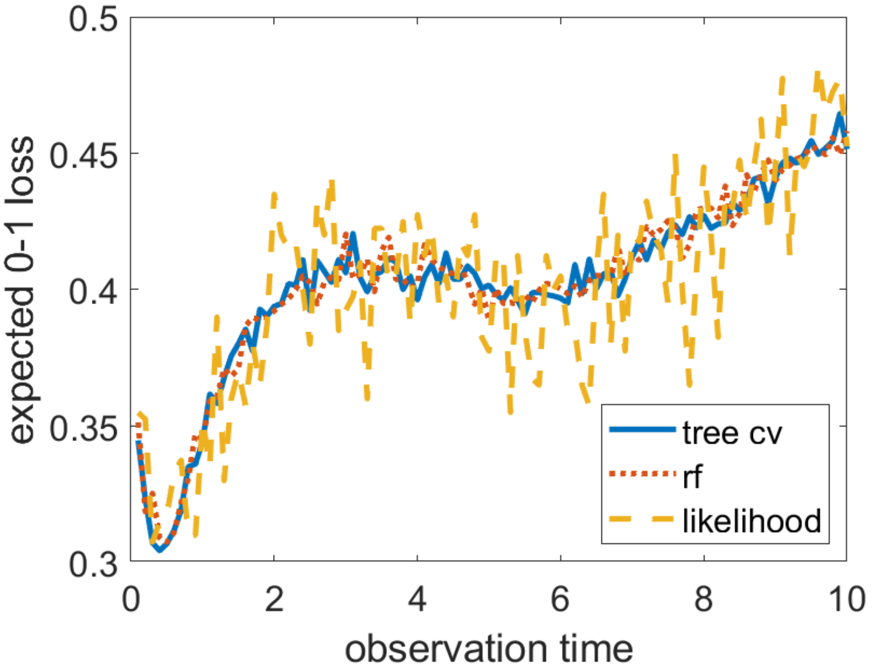


**Figure 3:** Plots of the approximated expected 0–1 loss functions produced by the tree classification approach with cross-classification (solid), the random forest classification approach (dotted), and the likelihood-based approach using a Laplace-type approximation to the marginal likelihood (dashed) for the infectious disease example with two models.

for comparison. The marginal likelihoods are computed using the generalised Gauss-Hermite quadrature approximation (5.7) with $Q = 30$ quadrature points for the death model and up to $Q = 30^2$ quadrature points for the SI model.

Figure 4 shows that for lower-dimensional designs all methods lead to designs with a very similar classification accuracy as measured by the distribution of the posterior model probabilities of the true model. For the higher-dimensional designs, Figure 5 indicates that the designs found using random forests are performing slightly better than the designs found using cross-validated trees. This comes at the cost of a higher computing time.

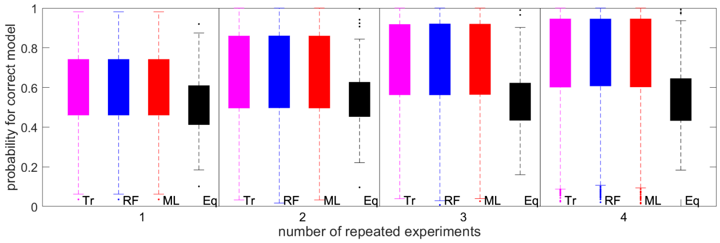


(a) $n_d = 1$

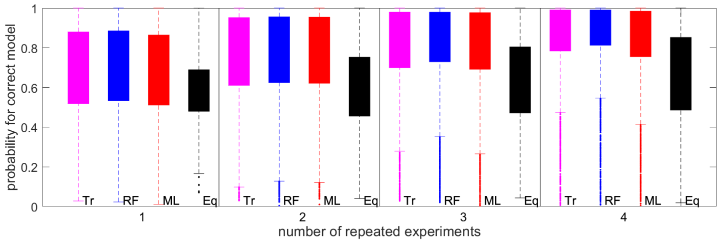


(b) $n_d = 2$

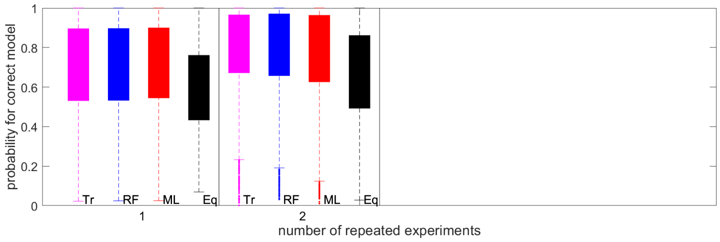


(c) $n_d = 3$

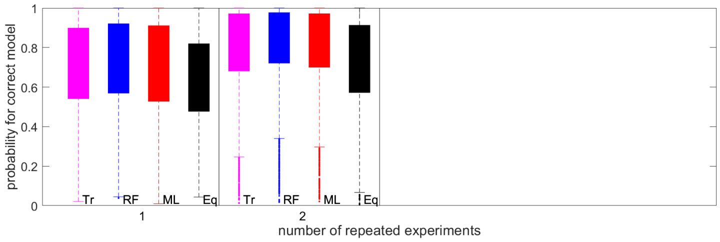


(d) $n_d = 4$

**Figure 4:** Distributions of posterior model probabilities of the correct model for 2K prior predictive simulations (1K from each of the two models) for the infectious disease example with two models. The data are all simulated at the respective optimal designs for the different approaches. The 0–1 loss is used as criterion. Settings with $q = 1$ to $q = 4$ realisations and $n_d = 1$ to $n_d = 4$ observations per realisation are considered ($q \leq 2$ for $n_d = 3$ and $n_d = 4$). For each setting, from left to right the boxplots are for the cross-validated tree classification design (Tr; magenta), the random forest classification design (RF; blue), the design found using the Laplace approximations to the marginal likelihoods (ML; red), and the equispaced design (Eq; black).

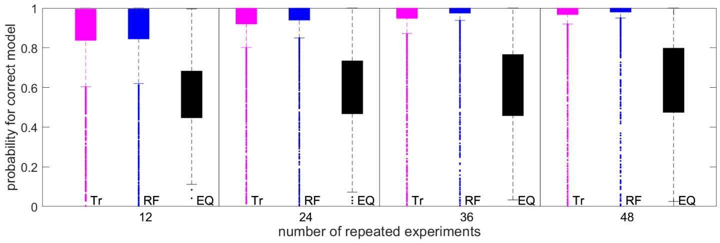


(a) $n_d = 1$

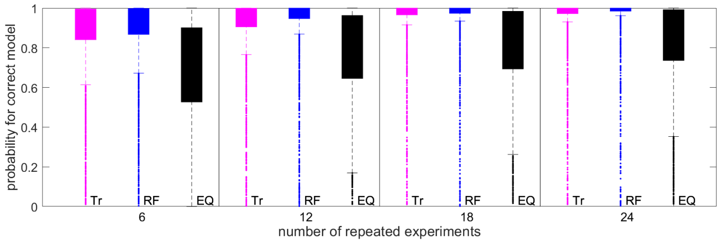


(b) $n_d = 2$

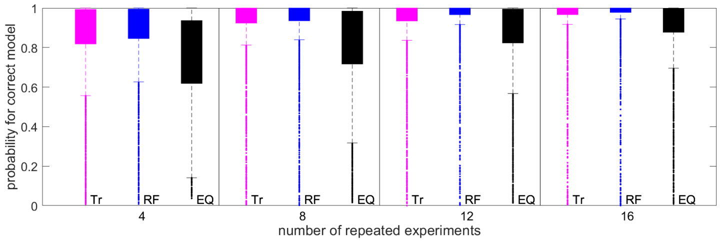


(c) $n_d = 3$

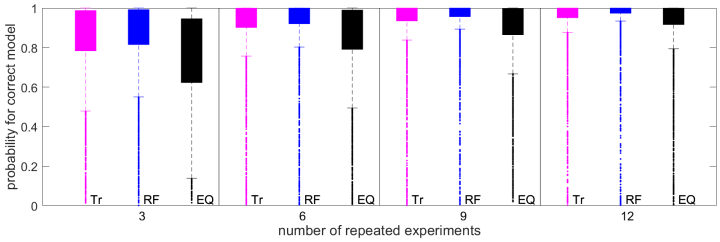


(d) $n_d = 4$

**Figure 5:** Distributions of posterior model probabilities of the correct model for 2K prior predictive simulations (1K from each of the two models) for the infectious disease example with two models. The data are all simulated at the respective optimal designs for the different approaches. The 0–1 loss is used as criterion. Settings with various numbers of realisations and $1 \leq n_d \leq 4$ observations per realisation are considered. The number of realisations were chosen such that the total number of observations $n = q \cdot n_d$ is equal to $n = 12$, 24, 36, or 48. For each setting, from left to right the boxplots are for the cross-validated tree classification design (Tr; magenta), the random forest classification design (RF; blue), and the equispaced design (Eq; black).

# 6 Additional Details and Results for Macrophage Example

## 6.1 Models and Prior Distributions

In all three models, a macrophage can acquire a new bacterium with a constant rate $\phi$ while there is no antibiotic in the medium ($t < t_{exp}$); this rate then drops to 0 for the remainder of the simulations. In model 1, we assume that a proportion $p > 0$ of available bacteria are non-replicating, so these are acquired by macrophages at rate $\phi\, p$, while replicating bacteria are acquired at rate $\phi(1-p)$. Intracellular bacteria are degraded at rate $d$ for replicating bacteria and rate $\epsilon$ for non-replicating bacteria. Within permissive macrophages containing $R > 1$ replicating bacteria, the number of replicating bacteria increases by one every time one of these bacteria divides, but this division rate is assumed to be a decreasing function of $R$ (due to limited resources for bacterial growth within a macrophage), expressed as $a\, e^{-bR}$, where $a$ is the maximum division rate of bacteria and $b$ is a dimensionless scaling parameter. Finally, in model (1), replicating bacteria within permissive macrophages become non-replicating at rate $\delta$. All these transitions are listed in Table 7.

**Table 7:** Three competing models considered in the macrophage example. $R(t)$ represents the number of replicating bacteria and $D(t)$ the number of non-replicating bacteria within a macrophage. In model 2, a proportion $q$ of macrophages are refractory and $1-q$ permissive.

| Model Number | Event Type | Update | Rate |
|---|---|---|---|
| (1) | Acquisition of R | $R(t)+1$ | $\phi(1-p)$ |
| | Acquisition of D | $D(t)+1$ | $\phi p$ |
| | Division | $R(t)+1$ | $a\, e^{-b\, R(t)}\, R(t)$ |
| | Loss of R | $R(t)-1$ | $d\, R(t)$ |
| | Loss of D | $D(t)-1$ | $\epsilon\, D(t)$ |
| | Switch of R to D | $R(t)-1,\ D(t)+1$ | $\delta\, R(t)$ |
| (2) Refractory | Acquisition of D | $D(t)+1$ | $\phi$ |
| | Loss of D | $D(t)-1$ | $\epsilon\, D(t)$ |
| (2) Permissive | Acquisition of R | $R(t)+1$ | $\phi$ |
| | Loss of R | $R(t)-1$ | $d\, R(t)$ |
| | Division | $R(t)+1$ | $a\, e^{-b\, R(t)}\, R(t)$ |
| (3) | Acquisition of R | $R(t)+1$ | $\phi$ |
| | Loss of R | $R(t)-1$ | $d\, R(t)$ |
| | Division | $R(t)+1$ | $a\, e^{-b\, R(t)}\, R(t)$ |

For each macrophage, numerical simulations of the three models are produced using the Gillespie algorithm (Gillespie, 1977). In line with the general experimental setup,

each macrophage is initially uninfected, but in model 2 it has a probability $q$ of being refractory. This state is set at the start of each simulation and does not change thereafter. To reproduce the data collection process described above, we produce two independent sets of simulations for each observation time $t_{obs}$ in a given experimental design. First, we run $S$ simulations of individual macrophages and record the proportion $\pi(t_{obs})$ of infected macrophages. Second, we run another set of simulations for the same duration until $S$ infected macrophages are obtained, from which we record the proportions $\{\mu_k(t_{obs}), k > 0\}$ of infected macrophages containing $k$ bacteria. This can be repeated multiple times to generate multiple sets of observations from each model $m$, parameter vector $\boldsymbol{\theta}_m$ and experimental design $\boldsymbol{d}$. Importantly, the simulations' results do not distinguish between replicating and non-replicating bacteria (model 1) or between refractory and permissive macrophages (model 2), as these cannot be told apart by microscopy alone.

The number of infected macrophages at time $t_{obs}$ has the binomial distribution $\mathrm{Bin}(S;\, \mathrm{E}[\pi(t_{obs})])$. Likewise, the vector of numbers of infected macrophages containing $k = 1, \ldots, K_+$ bacteria has the multinomial distribution
$\mathrm{Mult}(S; \{\mathrm{E}[\mu_1(t_{obs})], \ldots, \mathrm{E}[\mu_{K_+}(t_{obs})]\})$. The last category $K_+$ contains all macrophages with at least $K_+$ bacteria.

The most involved part is to obtain the expected proportions $\mathrm{E}[\pi(t_{obs})]$ and $\mathrm{E}[\mu_1(t_{obs})], \ldots, \mathrm{E}[\mu_{K_+}(t_{obs})]$ for any particular set of parameters. A system of linear differential equations consisting of the Kolmogorov forward equations for the models in Table 7 has to be solved to determine the expected proportions of macrophages that contain a certain number of replicating and non-replicating bacteria (see Restif et al. (2012)). The solution of this system can be computed using matrix exponentials. Considering only the total number of bacteria in a macrophage, the expected proportions $\mathrm{E}[\pi(t_{obs})], \mathrm{E}[\mu_1(t_{obs})], \ldots, \mathrm{E}[\mu_{K_+}(t_{obs})]$ can then be derived.

The prior distributions for each model were driven by the analysis of the experimental system in Restif et al. (2012). We assume truncated multivariate normal distributions, where the mean vector and variance-covariance matrix are based on the maximum likelihood estimates (MLEs) and the inverse of the Hessian obtained from the optimisation routine, respectively. All parameters are truncated below at 0. The proportion parameters $p$ and $q$ are additionally truncated above at 1.

In model 1, all macrophages are permissive, so $q = 0$. The mean vector and the variance-covariance matrix of the truncated normal prior for the remaining parameters of model 1 are given by

$$\boldsymbol{\mu}_1^\top = \begin{pmatrix} \overset{a}{6.46} & \overset{b}{1.54} & \overset{d}{0.073} & \overset{\delta}{2.529 \cdot 10^{-10}} & \overset{\epsilon}{0.035} & \overset{p}{0.097} & \overset{\phi}{0.25} \end{pmatrix}$$

and

$$\boldsymbol{\Sigma}_1 = \begin{array}{c} \\ a \\ b \\ d \\ \delta \\ \epsilon \\ p \\ \phi \end{array} \begin{array}{c} \begin{array}{ccccccc} a & b & d & \delta & \epsilon & p & \phi \end{array} \\ \begin{pmatrix} 32.8310 & & & & & & \\ 0.6224 & 0.0696 & & & & & \\ 0.1991 & -0.0017 & 0.0487 & & & & \\ 0.1258 & 0.0218 & -0.0164 & 0.0153 & & & \\ 0.0166 & 0.0048 & -0.0069 & 0.0052 & 0.0024 & & \\ 0.2142 & 0.0252 & -0.0061 & 0.0102 & 0.0039 & 0.0192 & \\ -0.0101 & 0.0001 & -0.0029 & 0.0018 & 0.0011 & 0.0018 & 0.0030 \end{pmatrix} \end{array}.$$

(The upper triangular part of the variance-covariance matrices is omitted.)

For model 2, where all bacteria are replicating and hence $\delta = p = 0$, the mean vector and the variance-covariance matrix are selected to be

$$\boldsymbol{\mu}_2^\top = \begin{array}{c} \begin{array}{cccccc} a & b & d & \epsilon & \phi & q \end{array} \\ (8.54221 \quad 1.450254 \quad 0.09111 \quad 0.03 \quad 0.25948 \quad 0.266837) \end{array}$$

and

$$\boldsymbol{\Sigma}_2 = \begin{array}{c} \\ a \\ b \\ d \\ \epsilon \\ \phi \\ q \end{array} \begin{array}{c} \begin{array}{cccccc} a & b & d & \epsilon & \phi & q \end{array} \\ \begin{pmatrix} 33.5250 & & & & & \\ 1.1380 & 0.3586 & & & & \\ 0.8252 & -0.1213 & 0.0952 & & & \\ 0.0253 & 0.0077 & -0.0023 & 0.1067 & & \\ -0.1471 & -0.0511 & 0.0197 & -0.0001 & 0.0355 & \\ 0.9048 & 0.1962 & -0.0658 & 0.0097 & -0.0284 & 0.2765 \end{pmatrix} \end{array}.$$

Finally, model 3 assumes that all macrophages are permissive and all bacteria are replicating, so $\delta = \epsilon = p = q = 0$. For this model the truncated normal prior's mean vector and variance-covariance matrix are

$$\boldsymbol{\mu}_3^\top = \begin{array}{c} \begin{array}{cccc} a & b & d & \phi \end{array} \\ (0.8161965 \quad 0.52672325 \quad 0.20740975 \quad 0.3203258) \end{array}$$

and

$$
\boldsymbol{\Sigma}_3 = \begin{array}{c} \\ a \\ b \\ d \\ \phi \end{array} \begin{array}{c} \begin{array}{cccc} a & b & d & \phi \end{array} \\ \begin{pmatrix} 0.7518 & & & \\ 0.1172 & 0.0506 & & \\ 0.0720 & -0.0090 & 0.0228 & \\ 0.0008 & -0.0106 & 0.0100 & 0.0287 \end{pmatrix} \end{array}.
$$

## 6.2 Optimal Designs

Tables 8 and 9 show the optimal designs for each classification method and for the different numbers of observation times. The tree and the random forest classification approaches lead to very similar designs.

**Table 8:** Optimal classification designs $(t_{exp}; \boldsymbol{t}_{obs})$ using trees or random forests under the 0–1 loss and equispaced designs for the macrophage model ($n$ = 1, 2, and 3).

| Method | $n = 1$ | | $n = 2$ | | | $n = 3$ | | | |
|---|---|---|---|---|---|---|---|---|---|
| | $t_{exp}$ | $\boldsymbol{t}_{obs}$ | $t_{exp}$ | $\boldsymbol{t}_{obs}$ | | $t_{exp}$ | $\boldsymbol{t}_{obs}$ | | |
| Tree | 1.20 | 10.00 | 0.09 | 1.75 | 10.00 | 0.09 | 1.25 | 2.75 | 10.00 |
| RF | 1.11 | 10.00 | 0.10 | 1.75 | 10.00 | 0.10 | 1.50 | 10.00 | 10.00 |
| Equi | 0.80 | 5.00 | 0.80 | 3.33 | 6.67 | 0.80 | 2.50 | 5.00 | 7.50 |

**Table 9:** Optimal classification designs $(t_{exp}; \boldsymbol{t}_{obs})$ using trees or random forests under the 0–1 loss and equispaced designs for the macrophage model ($n$ = 4 and 5).

| Method | $n = 4$ | | | | | $n = 5$ | | | | | |
|---|---|---|---|---|---|---|---|---|---|---|---|
| | $t_{exp}$ | $\boldsymbol{t}_{obs}$ | | | | $t_{exp}$ | $\boldsymbol{t}_{obs}$ | | | | |
| Tree | 0.09 | 0.75 | 2.25 | 9.00 | 10.00 | 0.10 | 0.75 | 2.50 | 2.75 | 10.00 | 10.00 |
| RF | 0.10 | 1.25 | 2.75 | 10.00 | 10.00 | 0.09 | 1.50 | 2.50 | 9.75 | 10.00 | 10.00 |
| Equi | 0.80 | 2.00 | 4.00 | 6.00 | 8.00 | 0.80 | 1.67 | 3.33 | 5.00 | 6.67 | 8.33 |

## 6.3 Misclassification Matrix

We can use the same random forest classifiers and their associated samples that were created to estimate the misclassification error rates in Table 4 of the main paper to compute the misclassification matrices. The misclassification matrices for the optimal designs obtained under the random forest classification approach are displayed in Figure 6. The classification power is very high for all the models. One can see that it is slightly more difficult to detect heterogeneity between bacteria (model 1) than heterogeneity between macrophages (model 2). The misclassification matrices for the designs obtained under the tree classification approach are almost identical.

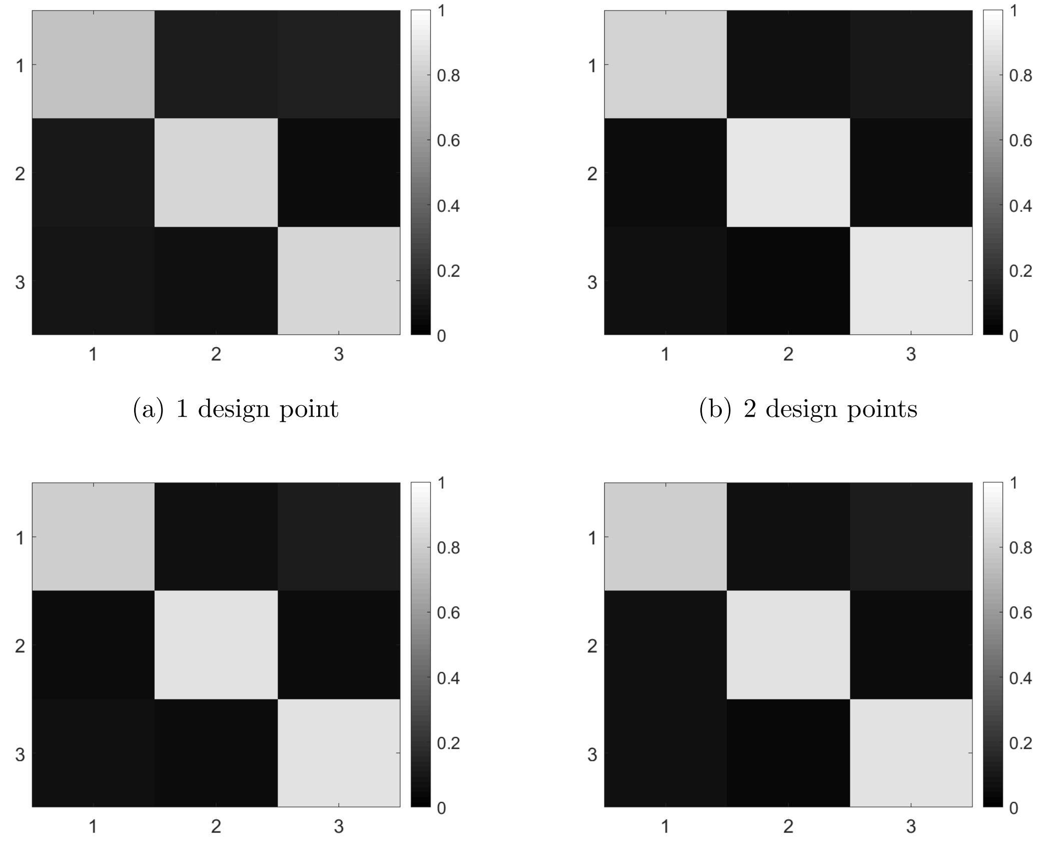


(a) 1 design point

(b) 2 design points

(c) 3 design points

(d) 4 design points

**Figure 6:** Misclassification matrices obtained for the *random forest classification designs* under the *0–1 loss* for the macrophage example. Designs for 1 – 4 observation times plus the exposure duration are considered.

# 7 Logistic Regression Example

We consider the logistic regression example of Overstall and Woods (2017) and Overstall et al. (2018). The response is binary, $y_{ij} \sim \mathcal{B}(p_{ij})$, and

$$\text{logit}(p_{ij}) = \beta_0 + \gamma_{0i} + \sum_{a=1}^{4} v_a(\beta_a + \gamma_{ai})x_{aij},$$

where $j = 1, \ldots, n_G$ and $i = 1, \ldots, G$. Here $G$ is the total number of groups and $n_G$ is the number of observations per group. The total number of observations is $n = G \times n_G$. The model parameter of interest is $\boldsymbol{\theta} = (\beta_0, \beta_1, \beta_2, \beta_3, \beta_4)^\top$. The random effect for the $i$th group is $\boldsymbol{\gamma}_i = (\gamma_{0i}, \gamma_{1i}, \gamma_{2i}, \gamma_{3i}, \gamma_{4i})^\top$. The observed vector of responses for the $i$th group is $\boldsymbol{y}_i = (y_{i1}, \ldots, y_{in_G})$ and the total dataset is denoted $\boldsymbol{y} = (\boldsymbol{y}_1, \ldots, \boldsymbol{y}_G)^\top$. The design vector is the concatenation of the controllable elements of the design matrix, $\boldsymbol{d} = \{x_{aij}; a = 1, \ldots, 4, i = 1, \ldots, G, j = 1, \ldots, n_G\}$ and is of length $n \times 4$. Each design element is restricted, $x_{aij} \in [-1, 1]$. The variable $v_a$ is an indicator variable that is equal to 1 if the $a$th predictor is present in the model. It may not be clear which of the four predictors should be included in the model, so there are $2^4 = 16$ possible models to choose from. We aim to select the design $\boldsymbol{d}$ that maximises our ability to discriminate between all possible models under various prior assumptions as described below.

As in Overstall et al. (2018), two different model structures are considered. The first structure is that all random effects (RE) are set to 0, resulting in the fixed effects (FE) structure. The second structure is that the random effects are allocated a distribution (RE structure). Within each chosen structure, there are 16 models to discriminate between. In both the FE and RE structures, we use the priors $\beta_0 \sim \mathcal{U}(-3, 3)$, $\beta_1 \sim \mathcal{U}(4, 10)$, $\beta_2 \sim \mathcal{U}(5, 11)$, $\beta_3 \sim \mathcal{U}(-6, 0)$, $\beta_4 \sim \mathcal{U}(-2.5, 3.5)$. We assume that all parameters are independent *a priori*. For the RE model we set $\gamma_{ai} \sim \mathcal{U}(-\zeta_a, \zeta_a)$ and allocate a triangular prior to $\zeta_a$, $p(\zeta_a) = 2(U_a - \zeta_a)/U_a^2$, $0 < \zeta_a < U_a$, where $(U_0, U_1, U_2, U_3, U_4) = (3, 3, 3, 1, 1)$. One possibility for the prior distribution placed on each model is a prior which depends on the number of predictors present in the model. Let $(v_{m1}, \ldots, v_{m4})$ denote the values of $(v_1, \ldots, v_4)$ for model $m$. A model prior accounting for Bayesian multiplicity (Scott and Berger, 2010) is

$$p(m) = \frac{1}{5\binom{4}{\sum_{a=1}^{4} v_{ma}}}. \tag{7.1}$$

In order to estimate the misclassification error rate under the Bayes classifier (the Bayes error rate) for some design $\boldsymbol{d}$, we need to estimate posterior model probabilities for $J$ datasets simulated from the prior predictive distributions of all the models. A common approach for rapid approximation of the evidence for model $m$, $p(\boldsymbol{y}|m, \boldsymbol{d})$, in the context of Bayesian optimal design is importance sampling (IS), where the importance distribution is the prior (e.g. Ryan et al. (2014)). However, if the data is informative (as might be the case in this example if $n$ is large), the number of IS samples to estimate

the evidence with reasonable precision may be prohibitively large. The situation is significantly worse for the RE structure, as an importance distribution is required over the space of both the parameter of interest and the random effects (see, e.g., Ryan et al. (2015)). For the FE structure and $n = 48$, using 100K importance samples from the prior and $J = 800$ (50 per model), the time taken to approximate the misclassification error rate for a random design on a cluster using 24 parallel threads was almost 2.75 minutes. This is very computationally intensive considering that we need to optimise over $48 \times 4$ design variables. Performing IS for the RE structure might be considered as completely intractable. Overstall et al. (2018) propose the use of normal-based approximations to the posterior in the Bayesian design context to provide a convenient estimate of the evidence. They consider the same logistic regression example but use normal priors to facilitate the approximation of the evidence. In some applications, a normal-based approximation may not be adequate.

In contrast, our classification approach avoids computing posterior quantities and requires only simulation from all the models. Interestingly, moving to the RE structure poses little additional difficulty for the classification approach as it remains trivial to simulate from the models. This is a significant advantage of the classification approach.

For the FE structure we consider $n \in \{6, 12, 24, 48\}$ and for the RE structure we consider $n_G = 6$ and $G \in 2, 4, 8$ (to give $n \in \{12, 24, 48\}$). Two prior distributions on the model indicator are trialled: (1) the prior where models are equally likely *a priori* and (2) the prior in (7.1) that corrects for Bayesian multiplicity. We refer to the first as the equal prior and the second as the unequal prior. For this example, the only design criterion that we consider is the misclassification error rate (the excepted 0–1 loss). During the design optimisation phase, we estimate the expected loss by employing cross-validated classification trees using a sample of size 80K (5K simulations per model). The observations are weighted within the trees according to their prior model probabilities. We consider a discretised design space for each $x_{aij}$ consisting of the five values $\{-1, -0.5, 0, 0.5, 1\}$.

After having obtained the optimal designs for the different scenarios regarding model structure (FE or RE) and prior distributions (equal or unequal), we attempt to assess the classification performance of these optimal designs using random forests. For each optimal design, 10K simulations under each model are used to train a random forest with 100 trees. A fresh set of $16 \times 10\text{K} = 160\text{K}$ simulations is used to estimate the misclassification error rate and the misclassification matrix. The model proportions of this test sample reflect the prior model probabilities. The results for the optimal designs of the different scenarios are shown in the rows with bold row labels in Table 10. For each scenario, results for optimal designs under different scenarios as well as a randomly generated design are also provided. For the randomly generated designs, each design point $x_{aij}$ equals 1 or $-1$ with equal probability.

The results suggest that the optimal designs found for this example are remarkably robust with respect to the assumed model structure (FE or RE) and the assumed prior

model probabilities (equal or unequal). The random design has the worst performance under all scenarios. We can also see a decrease in the misclassification error rate as the sample size is increased, as expected.

It is also of interest to see how well the optimal designs found under the tree classification approach perform in terms of posterior model probabilities. We conduct a simulation study under the FE structure using either the equal or the unequal prior. For each design we want to assess, we simulate a sample of 800 datasets from the marginal distributions of all the various models, where the proportion of datasets from a particular model in the sample corresponds to that model's prior model probability. For each of the 800 datasets, we approximate the posterior model probability of the model $m$ that generates the dataset $\boldsymbol{y}$ using IS with 100K prior simulations. As for the classification results in Table 10, we are also interested in the performance of optimal designs found under some wrongly assumed scenarios. We also consider a 'random' setup where we select designs randomly for each of the 800 datasets. Figure 7 shows the boxplots of the estimated posterior model probabilities of the correct model for some of the designs of interest when the true scenario is the FE structure with the equal prior. The resulting boxplots when the true scenario is the FE structure with the unequal prior are shown in Figure 8. It is again evident that the optimal designs found are robust under the choice of the structure (FE or RE) and the choice of the prior model probabilities (equal or unequal). We do not perform a simulation study under the RE structure given the increasing difficulty of estimating the posterior model probabilities under this structure.

It is important to note that the random forest-based validation results in Table 10 were obtained in a small fraction of the time that it took to conduct the simulation study used to produce the results in Figures 7 and 8.

Figures 9 and 10 show misclassification matrices for the logistic regression models under the FE structure for the equal and unequal priors, respectively. To produce the results, 10K simulations from each model are used to train a random forest with 100 trees. The misclassification matrices are then computed based on a fresh test dataset of size $16 \times 10K = 160K$ with model proportions in the dataset corresponding to the prior model probabilities (under the equal prior, 10K simulations are taken from each model). The improvement in classification accuracy is clear as the sample size is increased. When the unequal prior is selected, it is evident for small sample sizes that it is easier to classify the models with higher prior probability. The misclassification matrices for the RE structure are omitted because they are very similar.

**Table 10:** Shown are the misclassification error rates obtained at various optimal tree classification designs for the different logistic regression models. Four scenarios for the true model are considered: (1) FE structure under the equal prior, (2) FE structure under the unequal prior, (3) RE model under the equal prior and (4) RE model under the unequal prior. Rows with bold labels contain the results for the optimal designs under each scenario. Also shown, for each scenario, are the results for various designs obtained under different wrong scenarios and the results for a random design. The results suggest that the optimal designs found are robust to the model structure (FE or RE) and to the prior model probabilities (equal or unequal). The random design has the worst performance under all scenarios.

| FE structure under the equal prior | | | | |
|---|---|---|---|---|
| Design | Sample Size ($n$) | | | |
| | 6 | 12 | 24 | 48 |
| **FE equal** | 0.616 | 0.494 | 0.407 | 0.354 |
| FE unequal | 0.665 | 0.535 | 0.431 | 0.386 |
| RE equal | NA | 0.497 | 0.413 | 0.359 |
| random | 0.730 | 0.638 | 0.534 | 0.463 |

| FE structure under the unequal prior | | | | |
|---|---|---|---|---|
| Design | Sample Size ($n$) | | | |
| | 6 | 12 | 24 | 48 |
| FE equal | 0.511 | 0.416 | 0.337 | 0.290 |
| **FE unequal** | 0.480 | 0.409 | 0.340 | 0.307 |
| RE unequal | NA | 0.410 | 0.341 | 0.313 |
| random | 0.553 | 0.456 | 0.401 | 0.352 |

| RE model under the equal prior | | | | |
|---|---|---|---|---|
| Design | Sample Size ($n$) | | | |
| | 6 | 12 | 24 | 48 |
| FE equal | NA | 0.504 | 0.423 | 0.366 |
| **RE equal** | NA | 0.506 | 0.424 | 0.369 |
| RE unequal | NA | 0.545 | 0.442 | 0.399 |
| random | NA | 0.629 | 0.538 | 0.462 |

| RE model under the unequal prior | | | | |
|---|---|---|---|---|
| Design | Sample Size ($n$) | | | |
| | 6 | 12 | 24 | 48 |
| FE unequal | NA | 0.416 | 0.351 | 0.317 |
| RE equal | NA | 0.426 | 0.349 | 0.302 |
| **RE unequal** | NA | 0.416 | 0.349 | 0.316 |
| random | NA | 0.483 | 0.406 | 0.362 |

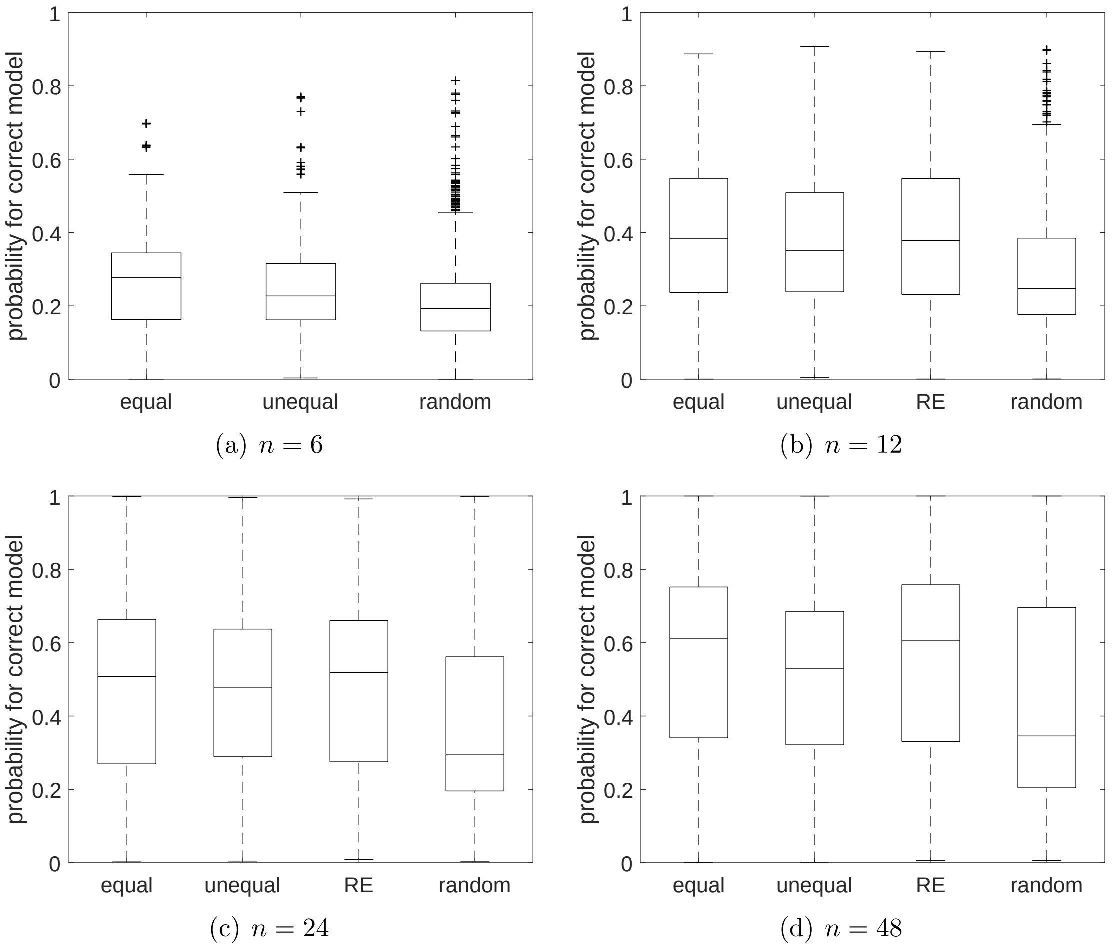


**Figure 7:** Estimated posterior model probabilities for the correct model by the validation study under the equal prior. Results based on sample sizes of (a) $n = 6$, (b) $n = 12$, (c) $n = 24$ and (d) $n = 48$. Several designs are considered: optimal design found under the correct (equal) prior, optimal design found under the wrong (unequal) prior, optimal design found under the wrong (RE) structure (no results for $n = 6$) and randomly selected designs.

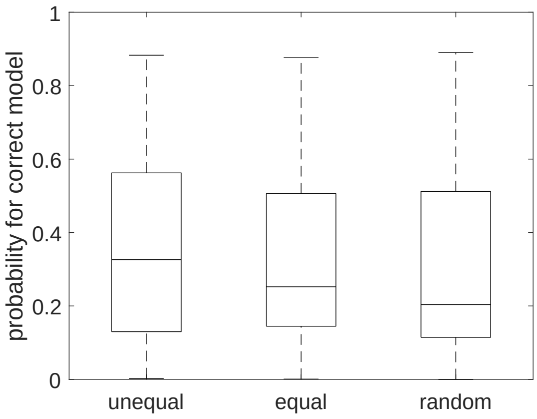


(a) $n = 6$

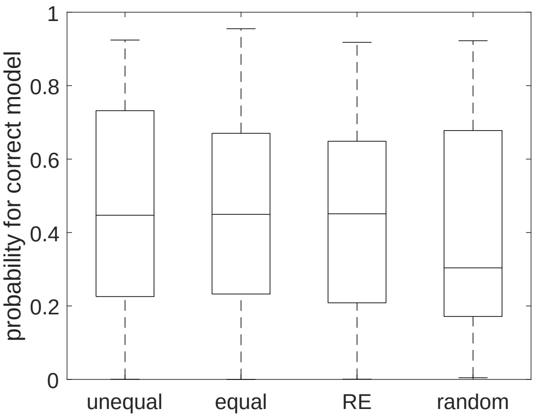


(b) $n = 12$

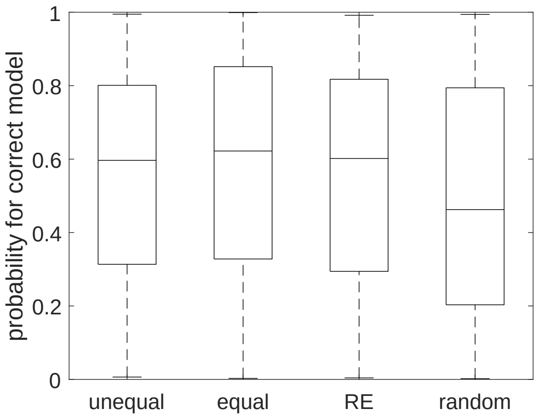


(c) $n = 24$

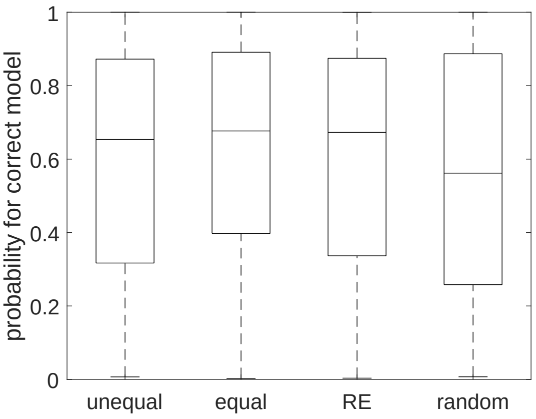


(d) $n = 48$

**Figure 8:** Estimated posterior model probabilities for the correct model by the validation study under the unequal prior. Results based on sample sizes of (a) $n = 6$, (b) $n = 12$, (c) $n = 24$ and (d) $n = 48$. Several designs are considered: optimal design found under the correct (unequal) prior, optimal design found under the wrong (equal) prior, optimal design found under the wrong (RE) structure (no results for $n = 6$) and randomly selected designs.

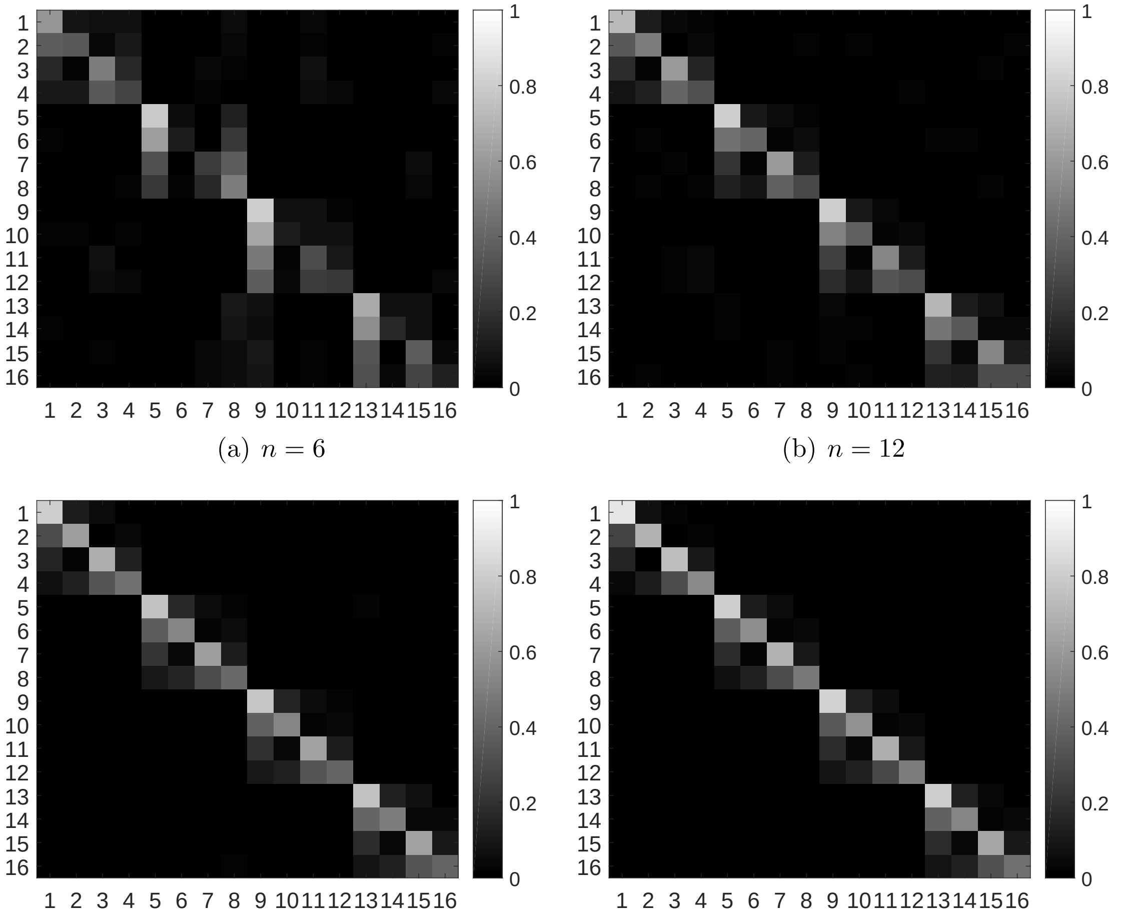

(a) $n = 6$ (b) $n = 12$

(c) $n = 24$ (d) $n = 48$

**Figure 9:** Misclassification matrices obtained for the *FE structures* of the logistic regression example with the *equal prior*.

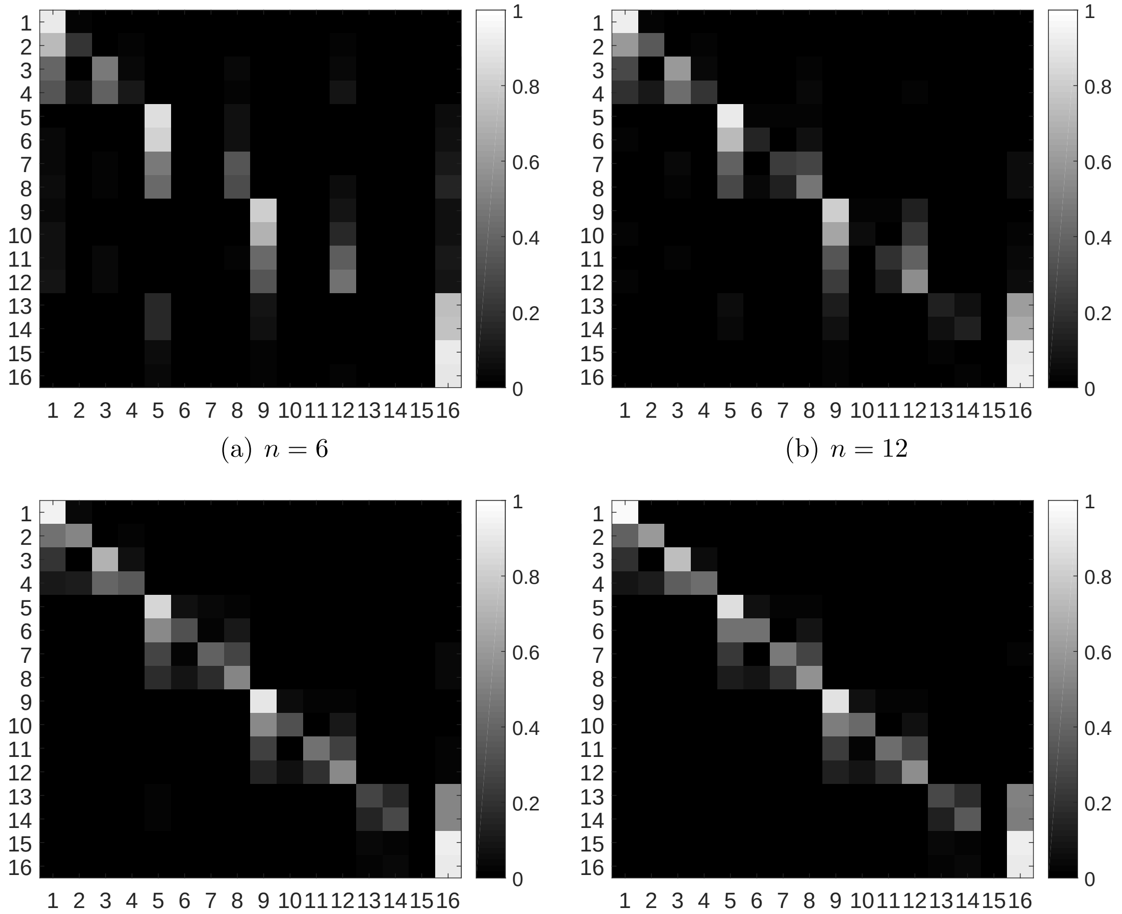

(a) $n = 6$ (b) $n = 12$ (c) $n = 24$ (d) $n = 48$

**Figure 10:** Misclassification matrices obtained for the *FE structures* of the logistic regression example with the *unequal prior*.

# 8 Spatial Extremes Example

In this example, the goal is to place a fixed number of measuring sites in space in order to maximise the ability to discriminate between different spatial models for extreme outcomes (e.g., maximum annual temperatures). There are many spatial models for extreme events, see Davison et al. (2012) for an overview. For this example, we consider to discriminate between three isotropic models: two *max-stable* models and one *copula* model.

## 8.1 Models

Max-stable processes are popular for modelling spatial extremes because they are the only possible limits of renormalised pointwise maxima of infinitely many independent copies of a stochastic process (de Haan and Ferreira, 2006). The advantage of working with the limiting process is that no knowledge about the underlying true process is necessary. Inference for extreme outcomes based on the true underlying process is fraught with high uncertainty and most often not feasible because only the tails of the distribution are observed. If the limiting assumption is (approximately) appropriate, it is much easier to model the extreme data according to a max-stable process.

All the univariate marginal distributions of a max-stable process are members of the family of *generalised extreme value (GEV)* distributions. We assume that all the univariate marginal distributions have a *unit Fréchet* distribution ($\Pr\{Y(\boldsymbol{x}) \leq y\} = \exp\{-1/y\}, y > 0$), so the focus is on modelling the dependence structure of the process. The assumption of unit Fréchet margins is not too restrictive from a practical perspective since a simple transformation can be applied to the univariate margins to make them unit Fréchet distributed, see Davison et al. (2012). The marginal parameters needed for that transformation can be estimated in a separate step. Alternatively, one may estimate the dependence and marginal parameters together.

The *spectral representation* of a max-stable process $\{Y(\boldsymbol{x}), \boldsymbol{x} \in \mathcal{X} \subseteq \mathbb{R}^d\}$ with unit Fréchet margins is given by

$$Y(\boldsymbol{x}) = \max_{i \geq 1} \varphi_i(\boldsymbol{x}), \quad \boldsymbol{x} \in \mathcal{X}, \tag{8.1}$$

where the *spectral functions* $\varphi_i(\boldsymbol{x}) = \zeta_i Z_i(\boldsymbol{x})$ are the products of the realisations $\{\zeta_i\}_{i=1}^{\infty}$ of a Poisson point process on the positive real line with intensity $d\Lambda(\zeta) = \zeta^{-2} d\zeta$ and of the independent realisations $\{Z_i(\boldsymbol{x}),\ \boldsymbol{x} \in \mathcal{X}\}_{i=1}^{\infty}$ of a non-negative stochastic process with continuous sample paths and $\mathrm{E}[Z(\boldsymbol{x})] = 1\ \forall\, \boldsymbol{x} \in \mathcal{X}$ (see, e.g., Ribatet (2013)).

Different max-stable processes are obtained by choosing different stochastic processes $Z$. We consider two very popular stationary models, the *extremal-t* model (Opitz,

2013) and the *Brown-Resnick* model with power variogram (Brown and Resnick, 1977; Kabluchko et al., 2009). The specifications for $Z_i(\boldsymbol{x})$ for each of the models are

$$\begin{aligned} &\text{Extremal-}t: && Z_i(\boldsymbol{x}) = \sqrt{\pi}\, 2^{-(\nu-2)/2}\, \Gamma\left\{(\nu+1)/2\right\}^{-1} \max\{0, \epsilon_i(\boldsymbol{x})\}^{\nu},\ \nu > 0, \\ &\text{Brown-Resnick:} && Z_i(\boldsymbol{x}) = \exp\left\{\varepsilon_i(\boldsymbol{x}) - \text{Var}[\varepsilon_i(\boldsymbol{x})]/2\right\}, \end{aligned}$$

where $\epsilon_i$ and $\varepsilon_i$ are independent copies of Gaussian processes.

In the case of the extremal-$t$ model, $\epsilon$ is a stationary Gaussian process defined by the correlation function $\rho(h)$, where $h$ is the Euclidean distance between two points. For our example, we assume the *powered exponential* or *stable* correlation function:

$$\rho(h) = \exp\left[-\left(h/\lambda\right)^{\kappa}\right], \quad \lambda > 0,\ 0 < \kappa \leq 2. \tag{8.2}$$

The Brown-Resnick process is defined by its semi-variogram. If the process $\varepsilon$ is a fractional Brownian motion centred at the origin, the Brown-Resnick process is stationary and the semi-variogram has the form

$$\gamma(h) = \left(h/\lambda\right)^{\kappa}, \quad \lambda > 0,\ 0 < \kappa \leq 2,$$

where $h$ denotes the distance between two locations.

Both models depend on two parameters governing the dependence between two locations separated by a distance $h$: the *range* parameter $\lambda$ and the *smoothness* parameter $\kappa$. In addition, the extremal-$t$ model has a *degrees of freedom* parameter denoted by $\nu$. We assume there is no discontinuity of the correlation function at $h = 0$ (i.e., no nugget effect).

The third model we consider is a copula model. Similar to the max-stable models, the univariate marginal distributions of the copula model are unit Fréchet. However, the extremal dependence between the locations is simply modelled by a standard (non-extremal) copula. For an introduction to copulas see Nelsen (2006). We assume the multivariate Student-$t$ copula in our example. The multivariate cumulative distribution function (CDF) at locations $(\boldsymbol{x}_1, \ldots, \boldsymbol{x}_H)$ implied by the *non-extremal Student-t copula* model (Demarta and McNeil, 2005) is

$$\Pr\{Y(\boldsymbol{x}_1) \leq y_1, \ldots, Y(\boldsymbol{x}_H) \leq y_H\} = T_{H;\nu}\{T_{1;\nu}^{-1}[F(y_1)], \ldots, T_{1;\nu}^{-1}[F(y_H)]; \boldsymbol{\Sigma}\},$$

where $F(y) = \exp\{-1/y\}$ is the CDF of the unit Fréchet distribution, $T_{1;\nu}^{-1}[\cdot]$ is the quantile function of the univariate Student-$t$ distribution with $\nu$ degrees of freedom, and $T_{H;\nu}\{\cdots; \boldsymbol{\Sigma}\}$ is the CDF of the $H$-variate Student-$t$ distribution with $\nu$ degrees of freedom and dispersion matrix $\boldsymbol{\Sigma}$. The diagonal elements of $\boldsymbol{\Sigma}$ are 1 and the off-diagonal elements contain the correlations between the locations. Therefore, the entries of $\boldsymbol{\Sigma}$ are given by $\boldsymbol{\Sigma}_{ij} = \rho(h_{ij})$ for $i, j = 1, \ldots, H$, where $h_{ij}$ is the distance between locations $i$ and $j$. As for the extremal-$t$ model, we assume the correlation function to be the powered exponential correlation function (8.2). This also implies that the non-extremal Student-$t$ copula model has the same set of parameters as the extremal-$t$ model: range ($\lambda$), smoothness ($\kappa$), and degrees of freedom ($\nu$).

## 8.2 Summary Statistics

If a reasonable amount of observations are collected at each location, the data collected quickly becomes very high-dimensional, while each observation is only marginally informative. This diminishes the classification power of the classifiers we use. We therefore aim to reduce the dimension of the data by generating informative summary statistics. Unfortunately, none of the statistics we consider guarantee consistent model choice. This can potentially result in large biases when estimating the posterior model probabilities (Robert et al., 2011), which can also affect the estimates of the misclassification error rates. However, trees and random forests work reasonably well with a sizeable amount of moderately informative feature variables. Therefore, we can include a wide variety of summary statistics, where each contains some information about the process. Considering the combined information of all the summary statistics, we expect that only a small loss in information is incurred compared to the full dataset.

First, we include all the *F-madogram* estimates for all the pairs of locations. The F-madogram (Cooley et al., 2006) is similar to the semi-variogram, but unlike the semi-variogram it also exists if the variances or means of the random variables are not finite. Given $n$ observations $\{y_1(\boldsymbol{x}_1), \ldots, y_n(\boldsymbol{x}_1)\}$ and $\{y_1(\boldsymbol{x}_2), \ldots, y_n(\boldsymbol{x}_2)\}$ collected at locations $\boldsymbol{x}_1$ as well as $\boldsymbol{x}_2$, the pairwise F-madogram between locations $\boldsymbol{x}_1$ and $\boldsymbol{x}_2$ is estimated as

$$\hat{\nu}_F(\boldsymbol{x}_1,\, \boldsymbol{x}_2) = \frac{1}{2n} \sum_{i=1}^{n} |F\{y_i(\boldsymbol{x}_1)\} - F\{y_i(\boldsymbol{x}_2)\}|,$$

where $F\{y\} = \exp\{-1/y\}$ is the CDF of the unit Fréchet distribution.

As a second set of summary statistics, we include estimates for all the pairwise *extremal coefficients* (Schlather and Tawn, 2003). For a max-stable process, the pairwise extremal coefficient between locations $\boldsymbol{x}_1$ and $\boldsymbol{x}_2$ is defined as the value $\theta(\boldsymbol{x}_1, \boldsymbol{x}_2)$ for which

$$\Pr(Y(\boldsymbol{x}_1) \leq y,\, Y(\boldsymbol{x}_2) \leq y) = \Pr(Y(\boldsymbol{x}_1) \leq y)^{\theta(\boldsymbol{x}_1, \boldsymbol{x}_2)} = \exp\left(-\frac{\theta(\boldsymbol{x}_1, \boldsymbol{x}_2)}{y}\right). \tag{8.3}$$

The pairwise extremal coefficient can assume values between 1 and 2. A value of $\theta(\boldsymbol{x}_1, \boldsymbol{x}_2) = 1$ indicates complete dependence between the two locations. If $\theta(\boldsymbol{x}_1, \boldsymbol{x}_2) = 2$, the two locations are completely independent. We estimate it using the fast estimator of Coles et al. (1999),

$$\hat{\theta}(\boldsymbol{x}_1, \boldsymbol{x}_2) = \frac{n}{\sum_{i=1}^{n} 1/\max\{y_i(\boldsymbol{x}_1), y_i(\boldsymbol{x}_2)\}}. \tag{8.4}$$

The extremal coefficient as defined by (8.3) only exists for max-stable processes. In general, the coefficient also depends on the level $y$. However, the quantities computed by Equation (8.4) might still provide useful information about the dependence structure.

For the $t$ copula model, Lee et al. (2018) demonstrate by simulation that the estimates given by (8.4) are indeed informative about the dependence structure.

The last set of summary statistics we consider is the set of *Kendall's* $\tau$ estimates between all pairs of locations. Kendall's $\tau$ between locations $\boldsymbol{x}_1$ and $\boldsymbol{x}_2$ is estimated by

$$\hat{\tau}(\boldsymbol{x}_1, \boldsymbol{x}_2) = \frac{2}{n\,(n-1)} \sum_{1 \leq i < j \leq n} \operatorname{sign}[y_i(\boldsymbol{x}_1) - y_j(\boldsymbol{x}_1)] \operatorname{sign}[y_i(\boldsymbol{x}_2) - y_j(\boldsymbol{x}_2)].$$

Dombry et al. (2018) show that for max-stable processes Kendall's $\tau$ is equal to the probability that the maxima at two locations occur concurrently and are therefore attained for the same extremal function, so

$$\tau(\boldsymbol{x}_1, \boldsymbol{x}_2) = \Pr\left(\arg\max_{i \geq 1} \varphi_i(\boldsymbol{x}_1) = \arg\max_{i \geq 1} \varphi_i(\boldsymbol{x}_2)\right).$$

All of the summary statistics we incorporate are also considered by Lee et al. (2018), who perform ABC model selection using the summary statistic projection method of Prangle et al. (2014) for a very similar set of models as in this example. Therefore, a more detailed discussion of the summary statistics can be found in Lee et al. (2018).

## 8.3 Bayesian Inference for Spatial Extremes Models

The likelihood functions of max-stable models are practically intractable for most models for dimensions greater than two or three. Composite likelihood methods have been the most popular way to conduct classical inference for max-stable models, so model discrimination is usually based on the composite likelihood information criterion (CLIC) (Padoan et al., 2010).

The observed extrema at several locations might occur at the same time, which means that the extrema at these locations arise from the same extremal function $\varphi_i(\boldsymbol{x})$ in Equation (8.1). The locations can then be partitioned according to which extremal functions $\varphi_i(\boldsymbol{x})$ produce the extreme observations at the different locations. Stephenson and Tawn (2005) show that the joint likelihood of the extreme observations and the partitions is substantially simpler than the likelihood of the extreme observations without knowledge of the partitions. Thibaud et al. (2016) and Dombry et al. (2017) use this property to devise a Gibbs sampler with the partitions as auxiliary variables to conduct Bayesian inference for max-stable models. However, even the augmented likelihoods are expensive to evaluate for the Brown-Resnick and extremal-$t$ model because they include multivariate Gaussian (Brown-Resnick) and Student-$t$ (extremal-$t$) CDFs.

Due to the intractability of the likelihoods, ABC has also been a popular method for Bayesian inference of max-stable models, see, e.g., Erhardt and Smith (2012) or the overview in Erhardt and Sisson (2015). Lee et al. (2018) present an ABC application with the joint goal of model selection and parameter estimation for the same set of

models we consider. Hainy et al. (2016) seek to find optimal designs for parameter estimation for the extremal-$t$ model with $\nu = 1$ (called the 'Schlather model'). They use ABC to estimate the posterior variances, which they use as design criterion. Their design algorithm is confined to very low-dimensional design spaces in order to be able to store the reference table for all possible designs. They sequentially select the best single location among a small set of possible locations. With our classification approach, we are able to overcome these limitations.

## 8.4 Settings and Results

In our example, we want to select $H$ ($H = 3, \ldots, 8$) locations on a regular grid such that the ability to discriminate between the three models as measured by the misclassification error rate is optimised. We search the $H$ optimal design points over a regular $6 \times 6$ grid laid over a square with edge length 10. The data consist of $n = 10$ independent realisations of the process collected at all the locations. Due to the isotropic nature of the processes, there are potentially many equivalent optimal solutions. With our modification of the coordinate exchange algorithm using 20 random starts, we seek to find one of these designs or at least a nearly optimal design.

We assume the following prior distributions:

$$\begin{aligned}\log(\lambda) &\sim \mathcal{N}(1,\, 4),\\ \kappa &\sim \mathcal{U}(0,\, 2),\\ \log(\nu) &\sim \mathcal{N}(0,\, 1) \text{ truncated on } [-2.5,\, 2.5].\end{aligned}$$

Furthermore, we assume equal prior model probabilities (= 1/3) for all models.

Simulating from the $t$ copula model is straightforward. It only requires simulating from a multivariate $t$ distribution and then transforming the margins with respect to the univariate $t$ CDF followed by the inverse unit Fréchet CDF. For simulating from the max-stable models, we use the exact simulation algorithm via extremal functions of Dombry et al. (2016).

During the design phase, we use cross-validated classification trees as well as random forests with 500 trees using out-of-bag class predictions to estimate the misclassification error rates. We had implemented the simulator functions for this example in R, therefore we use the R function `rpart` for classification trees, for which we keep all the default settings except for not considering any surrogate splits to speed up computing time. For random forests, we employ the function `randomForest` from the R (R Core Team, 2018) package of the same name (Liaw and Wiener, 2002). The simulated sets for both methods contain 5K simulations per model. The optimal designs obtained for these two methods are shown in Figures 11 (trees) and 12 (random forests).

To evaluate the designs found by our classification approach, we repeat estimating the misclassification error rate via random forests with 500 trees using out-of-bag class predictions on 100 different simulated samples of size 15K (5K simulations per model) from

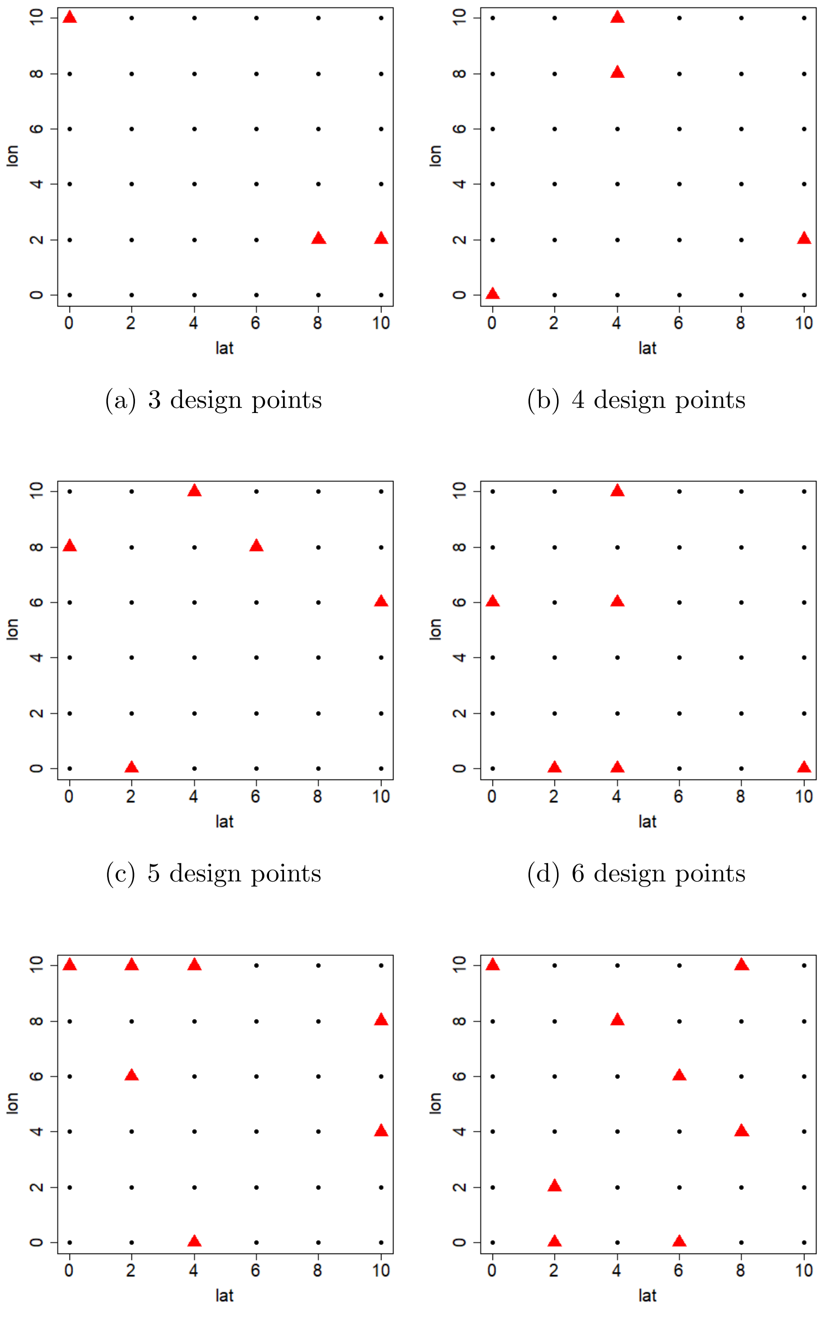


(a) 3 design points (b) 4 design points

(c) 5 design points (d) 6 design points

(e) 7 design points (f) 8 design points

**Figure 11:** Optimal classification designs found using *trees* for design sizes from three to eight for the spatial extremes example. Selected design points are marked by red triangles.

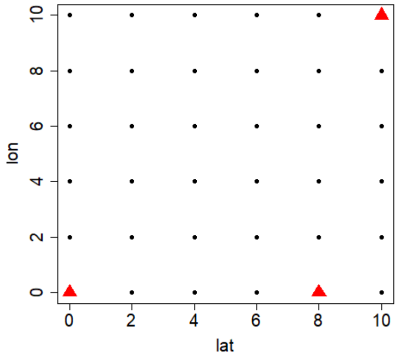


(a) 3 design points

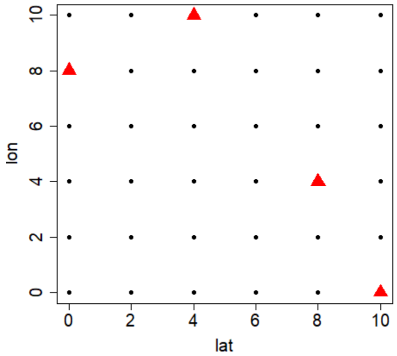


(b) 4 design points

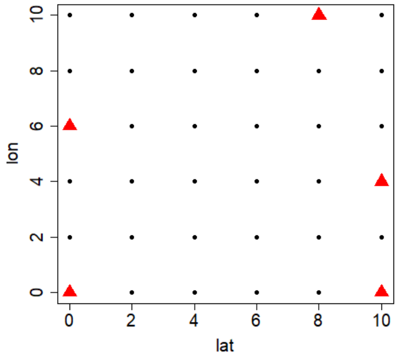


(c) 5 design points

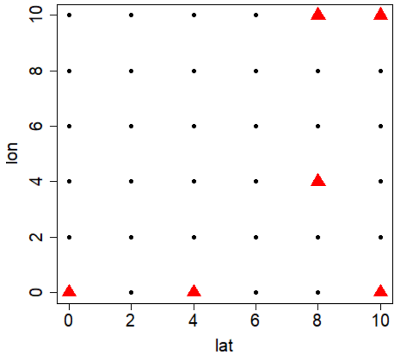


(d) 6 design points

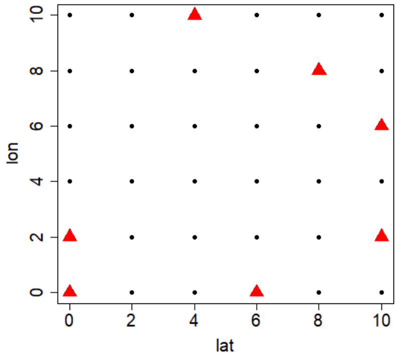


(e) 7 design points

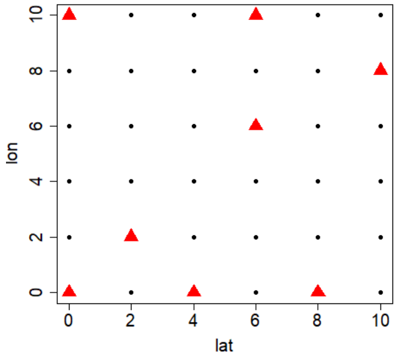


(f) 8 design points

**Figure 12:** Optimal classification designs found using *random forests* for design sizes from three to eight for the spatial extremes example. Selected design points are marked by red triangles.

the prior predictive distribution. The distributions of the estimated misclassification error rates are plotted in Figure 13. We also include the distributions of the estimated misclassification error rates for 100 simulated samples from the prior predictive distribution generated on 100 randomly selected designs. The optimal classification designs found using random forests clearly perform best for all design sizes. Using classification trees with cross-validation instead of random forests leads to designs which are a bit worse. However, the average misclassification error rate of the classification tree designs is still smaller than the average error rate of the random designs up until 7 design points.

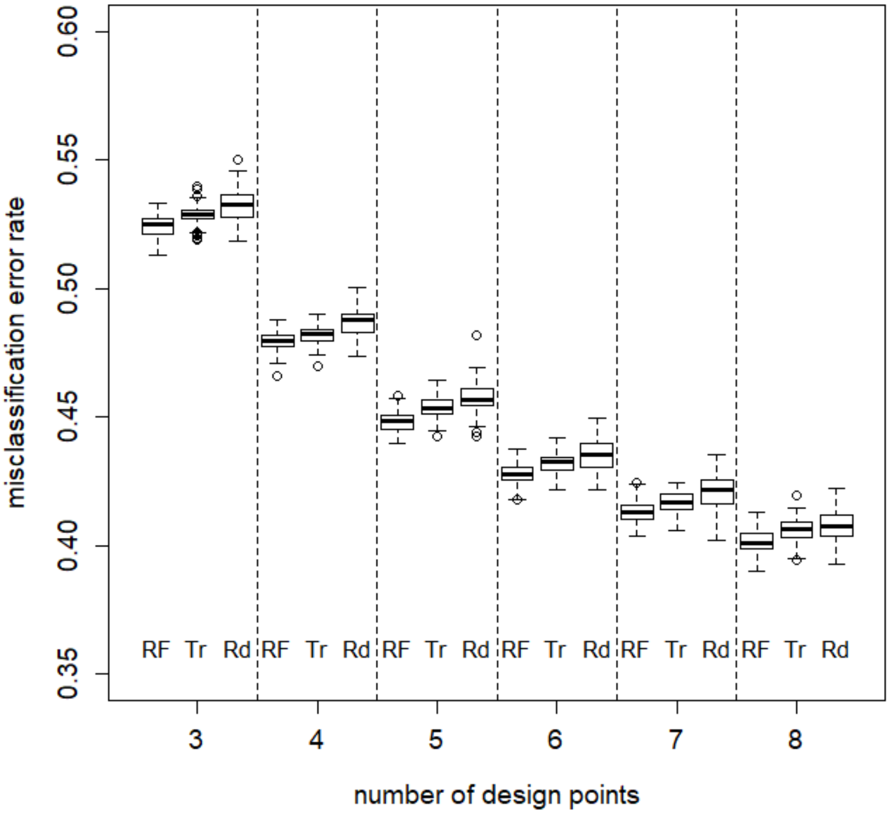


**Figure 13:** Spatial extremes example: distributions of the random forest-estimated misclassification error rates over 100 random samples of size 15K generated from the prior predictive distribution at the optimal classification designs found using random forests (rf) or trees (tr) for design sizes from three to eight. The distributions of the misclassification error rates over 100 random samples of size 15K generated from the prior predictive distribution at 100 random designs (rd) are also shown for the same design sizes.

In addition to the misclassification error rate, we also compute the misclassification matrix yielded by the random forest classifier for each of the 100 simulated samples for each evaluated design. The average misclassification matrices over the 100 samples are depicted in Figure 14 for the optimal designs obtained by the random forest classification approach. They show that discriminating between the two max-stable models is more difficult than discriminating between the $t$ copula model and either of the max-stable models.

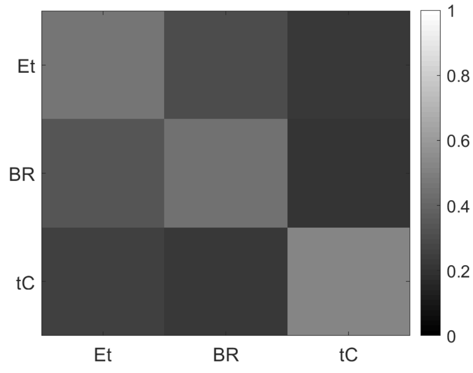


(a) 3 design points

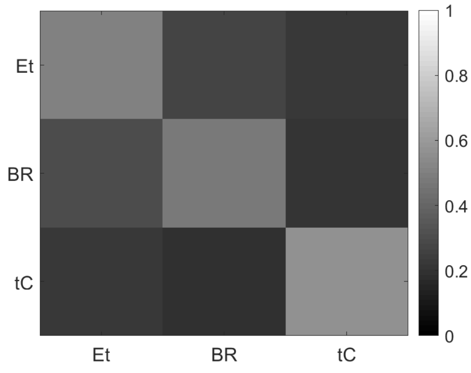


(b) 4 design points

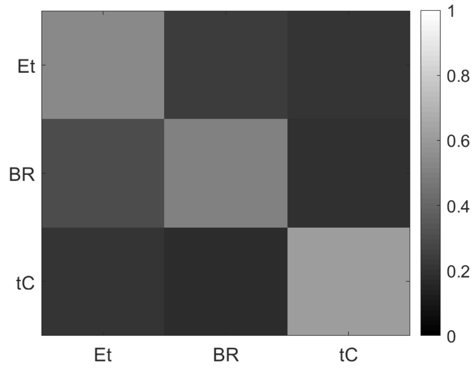


(c) 5 design points

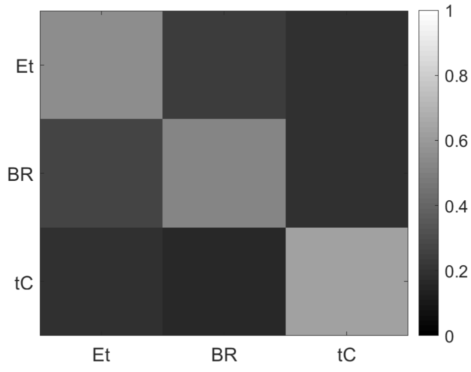


(d) 6 design points

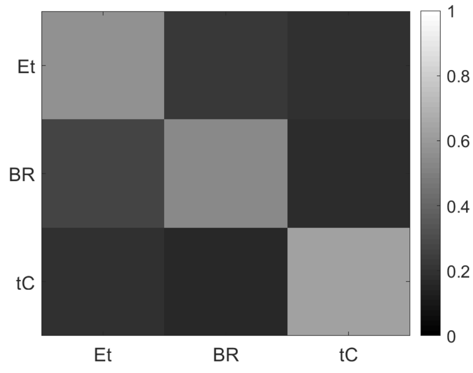


(e) 7 design points

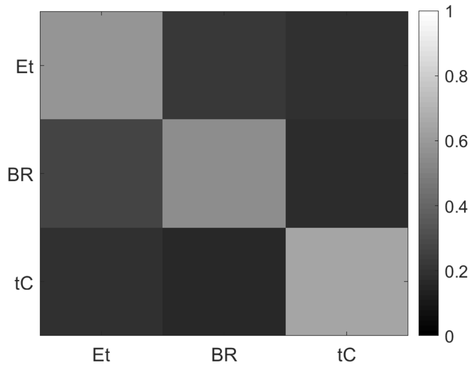


(f) 8 design points

**Figure 14:** Average misclassification matrices over 100 simulated prior predictive samples obtained for the *random forest classification designs* for the spatial extremes example. Design sizes from 3 – 8 design points are considered.

# References


Breiman, L. (1996). Bagging predictors. *Machine Learning*, 24(2):123–140.

Breiman, L. (2001). Random forests. *Machine Learning*, 45(1):5–32.

Breiman, L., Friedman, J., Olshen, R. A., and Stone, C. J. (1984). *Classification and Regression Trees*. Chapman & Hall/CRC, Boca Raton.

Brown, B. M. and Resnick, S. T. (1977). Extreme values of independent stochastic processes. *Journal of Applied Probability*, 14(4):732–739.

Coles, S., Heffernan, J., and Tawn, J. (1999). Dependence measures for extreme value analyses. *Extremes*, 2(4):339–365.

Cook, A. R., Gibson, G. J., and Gilligan, C. A. (2008). Optimal observation times in experimental epidemic processes. *Biometrics*, 64(3):860–868.

Cooley, D., Naveau, P., and Poncet, P. (2006). Variograms for spatial max-stable random fields. In Bertail, P., Soulier, P., and Doukhan, P., editors, *Dependence in Probability and Statistics*, pages 373–390. Springer, New York.

Cover, T. and Hart, P. (1967). Nearest neighbor pattern classification. *IEEE Transactions on Information Theory*, 13(1):21–27.

Davison, A. C., Padoan, S. A., and Ribatet, M. (2012). Statistical modeling of spatial extremes. *Statistical Science*, 27(2):161–186.

de Haan, L. and Ferreira, A. F. (2006). *Extreme Value Theory: An Introduction*. Springer, New York.

Demarta, S. and McNeil, A. J. (2005). The t copula and related copulas. *International Statistical Review*, 73(1):111–129.

Dombry, C., Engelke, S., and Oesting, M. (2016). Exact simulation of max-stable processes. *Biometrika*, 103(2):303–317.

Dombry, C., Engelke, S., and Oesting, M. (2017). Bayesian inference for multivariate extreme value distributions. *Electronic Journal of Statistics*, 11(2):4813–4844.

Dombry, C., Ribatet, M., and Stoev, S. (2018). Probabilities of concurrent extremes. *Journal of the American Statistical Association*, 113(524):1565–1582.

Elhay, S. and Kautsky, J. (1987). Algorithm 655: IQPACK, FORTRAN subroutines for the weights of interpolatory quadrature. *ACM Transactions on Mathematical Software*, 13(4):399–415.

Erhardt, R. J. and Sisson, S. A. (2015). Modelling extremes using approximate Bayesian computation. In Dey, D. K. and Yan, J., editors, *Extreme Value Modeling and Risk Analysis: Methods and Applications*, pages 281–306. Chapman and Hall/CRC.

Erhardt, R. J. and Smith, R. L. (2012). Approximate Bayesian computing for spatial extremes. *Computational Statistics & Data Analysis*, 56(6):1468–1481.

Gelman, A., Carlin, J. B., Stern, H. S., Dunson, D. B., Vehtari, A., and Rubin, D. B. (2013). *Bayesian Data Analysis*. Chapman and Hall/CRC, 3rd edition.

Gillespie, D. T. (1977). Exact stochastic simulation of coupled chemical reactions. *The Journal of Physical Chemistry*, 81(25):2340–2361.

Grimmett, G. R. and Stirzaker, D. R. (2001). *Probability and Random Processes*. Oxford University Press, New York, 3rd edition.

Hainy, M., Müller, W. G., and Wagner, H. (2016). Likelihood-free simulation-based optimal design with an application to spatial extremes. *Stochastic Environmental Research and Risk Assessment*, 30(2):481–492.

Hastie, T., Tibshirani, R., and Friedman, J. (2009). *The Elements of Statistical Learning*. Springer, New York.

Higham, N. J. (2008). *Functions of Matrices: Theory and Computation*. Society for Industrial and Applied Mathematics, Philadelphia.

Jäckel, P. (2005). A Note on Multivariate Gauss-Hermite Quadrature. https://pdfs.semanticscholar.org/0e39/32b776eb3803e0f0ae2e414f69399daa411d.pdf, downloaded 11$^{\text{th}}$ March 2019.

Kabluchko, Z., Schlather, M., and de Haan, L. (2009). Stationary max-stable fields associated to negative definite functions. *The Annals of Probability*, 37(5):2042–2065.

Kautsky, J. and Elhay, S. (1982). Calculation of the weights of interpolatory quadratures. *Numerische Mathematik*, 40:407–422.

Lee, X. J., Hainy, M., McKeone, J. P., Drovandi, C. C., and Pettitt, A. N. (2018). ABC model selection for spatial extremes models applied to South Australian maximum temperature data. *Computational Statistics & Data Analysis*, 128:128–144.

Liaw, A. and Wiener, M. (2002). Classification and regression by randomForest. *R News*, 2(3):18–22.

Nelder, J. A. and Mead, R. (1965). A simplex method for function minimization. *The Computer Journal*, 7(4):308–313.

Nelsen, R. B. (2006). *An Introduction to Copulas*. Springer, New York, 2nd edition.

Opitz, T. (2013). Extremal t processes: Elliptical domain of attraction and a spectral representation. *Journal of Multivariate Analysis*, 122:409–413.

Overstall, A. M., McGree, J. M., and Drovandi, C. C. (2018). An approach for finding fully Bayesian optimal designs using normal-based approximations to loss functions. *Statistics and Computing*, 28(2):343–358.

Overstall, A. M. and Woods, D. C. (2017). Bayesian design of experiments using approximate coordinate exchange. *Technometrics*, 59(4):458–470.

Padoan, S. A., Ribatet, M., and Sisson, S. A. (2010). Likelihood-based inference for max-stable processes. *Journal of the American Statistical Association*, 105(489):263–277.

Prangle, D., Fearnhead, P., Cox, M. P., Biggs, P. J., and French, N. P. (2014). Semi-automatic selection of summary statistics for ABC model choice. *Statistical Applications in Genetics and Molecular Biology*, 13(1):67–82.

R Core Team (2018). *R: A Language and Environment for Statistical Computing*. R Foundation for Statistical Computing, Vienna, Austria.

Rasmussen, C. E. and Williams, C. K. I. (2006). *Gaussian Processes for Machine Learning*. MIT Press, Cambridge.

Restif, O., Goh, Y. S., Palayret, M., Grant, A. J., McKinley, T. J., Clark, M. R., and Mastoeni, P. (2012). Quantification of the effects of antibodies on the extra- and intracellular dynamics of *Salmonella enterica*. *Journal of the Royal Society Interface*, 10(79).

Ribatet, M. (2013). Spatial extremes: max-stable processes at work. *Journal de la Société Française de Statistique*, 154(2):156–177.

Robert, C. P., Cornuet, J.-M., Marin, J.-M., and Pillai, N. S. (2011). Lack of confidence in approximate Bayesian computation model choice. *Proceedings of the National Academy of Sciences of the USA*, 108(37):15112–15117.

Ryan, E. G., Drovandi, C. C., and Pettitt, A. N. (2015). Simulation-based fully Bayesian experimental design for mixed effects models. *Computational Statistics & Data Analysis*, 92:26–39.

Ryan, E. G., Drovandi, C. C., Thompson, M. H., and Pettitt, A. N. (2014). Towards Bayesian experimental design for nonlinear models that require a large number of sampling times. *Computational Statistics & Data Analysis*, 70:45–60.

Schlather, M. and Tawn, J. A. (2003). A dependence measure for multivariate and spatial extreme values: properties and inference. *Biometrika*, 90(1):139–156.

Scott, J. G. and Berger, J. O. (2010). Bayes and empirical-Bayes multiplicity adjustment in the variable-selection problem. *The Annals of Statistics*, 38(5):2587–2619.

Stephenson, A. and Tawn, J. (2005). Exploiting occurrence times in likelihood inference for componentwise maxima. *Biometrika*, 92(1):213–227.

Thibaud, E., Aalto, J., Cooley, D. S., Davison, A. C., and Heikkinen, J. (2016). Bayesian inference for the Brown-Resnick process, with an application to extreme low temperatures. *The Annals of Applied Statistics*, 10(4):2303–2324.
